# Evaluating the utility of brightfield image data for mechanism of action prediction

**DOI:** 10.1371/journal.pcbi.1011323

**Published:** 2023-07-25

**Authors:** Philip John Harrison, Ankit Gupta, Jonne Rietdijk, Håkan Wieslander, Jordi Carreras-Puigvert, Polina Georgiev, Carolina Wählby, Ola Spjuth, Ida-Maria Sintorn

**Affiliations:** 1 Department of Pharmaceutical Biosciences, Uppsala University, Uppsala, Sweden; 2 Science for Life Laboratory, Uppsala, Sweden; 3 Department of Information Technology, Uppsala University, Uppsala, Sweden; University of Cambridge, UNITED KINGDOM

## Abstract

Fluorescence staining techniques, such as Cell Painting, together with fluorescence microscopy have proven invaluable for visualizing and quantifying the effects that drugs and other perturbations have on cultured cells. However, fluorescence microscopy is expensive, time-consuming, labor-intensive, and the stains applied can be cytotoxic, interfering with the activity under study. The simplest form of microscopy, brightfield microscopy, lacks these downsides, but the images produced have low contrast and the cellular compartments are difficult to discern. Nevertheless, by harnessing deep learning, these brightfield images may still be sufficient for various predictive purposes. In this study, we compared the predictive performance of models trained on fluorescence images to those trained on brightfield images for predicting the mechanism of action (MoA) of different drugs. We also extracted CellProfiler features from the fluorescence images and used them to benchmark the performance. Overall, we found comparable and largely correlated predictive performance for the two imaging modalities. This is promising for future studies of MoAs in time-lapse experiments for which using fluorescence images is problematic. Explorations based on explainable AI techniques also provided valuable insights regarding compounds that were better predicted by one modality over the other.

## Author summary

In our work we provide a thorough comparison of deep learning models trained on fluorescence images versus those trained on brightfield images for the purpose of mechanism of action prediction. Fluorescence microscopy, which uses fluorescent dyes to stain specific cellular compartments, is an invaluable method in image cytometry but it is time-consuming, expensive, labour intensive, and the dyes used can be toxic to the cells. Brightfield microscopy, the simplest form of light microscopy, lacks these downsides but the images produced do not have the clear contrast of the cellular compartments. We show that with brightfield images it is possible to obtain very similar predictive performance, and in some cases superior performance, due to the brightfield images containing additional information not available in the fluorescence images. We validate these claims using explainable AI techniques. This competitive performance of the models based on brightfield is very promising for live cell time-lapse experiments for which using fluorescence microscopy is problematic especially due to the toxicity of some of the dyes.

## Introduction

Mechanism of action (MoA) describes the biological process by which a compound exhibits a pharmacological effect, such as the proteins targeted or the pathways modulated. Establishing a compound’s MoA provides particularly useful information for lead compounds prior to clinical trials and for identifying potentially adverse or toxic effects [1]. Various assays can be used to provide information on a compound’s MoA, including transcriptomics, proteomics and metabolomics assays [1]. Recently, morphology-based high-content imaging assays have proven beneficial for this task [2] and are also significantly easier and less expensive to scale to high-throughput than other assay types [3].

The simplest, cheapest, and least invasive form of light microscopy is brightfield (BF) microscopy. Due to the thinness and transparency of most cells, BF images typically have low contrast, making it difficult to detect internal cell structures. To overcome this limitation fluorescent dyes can be used. Fluorescence (FL) microscopy uses fluorescent dyes to stain specific targets (e.g. cell compartments) within the sample [4]. A noteworthy FL-based protocol is the Cell Painting assay [5] which combines six different stains to highlight eight different sub-cellular compartments. However, FL imaging is much more labor-intensive and expensive than BF imaging and is often not suitable for staining living cells. Live-cell compatible stains or fluorescently tagged proteins can be used for live cell staining, however the dyes required for imaging some of the cellular components can be toxic to the cells, and this toxicity becomes amplified with multiple imaging exposures [6]. Another more pervasive phototoxic effect in FL microscopy is photobleaching which not only decreases the fluorescent signal but also releases free-radicals [7].

Traditional analysis pipelines in image cytometry follow the path of identifying, segmenting, and extracting handcrafted quantitative features from the cells, often using the CellProfiler (CP) [8] software package. Common features include those related to size, shape, pixel intensity, and texture. For fluorescent images, these features can be extracted at the level of the various cellular compartments. These features can then be used as input to machine learning models. This approach has been successfully applied to MoA prediction for cells perturbed by small molecules [2, 9, 10] and to group genes based on functional similarity [11]. As an alternative approach, one can use deep learning methods [12], specifically convolutional neural networks (CNNs), to perform the predictive task directly from the raw pixel intensity data in an end-to-end data-driven fashion, circumventing the need for cell segmentation and determining which features to extract [13]. This approach has proven successful for MoA prediction [14].

**Fig 1:**
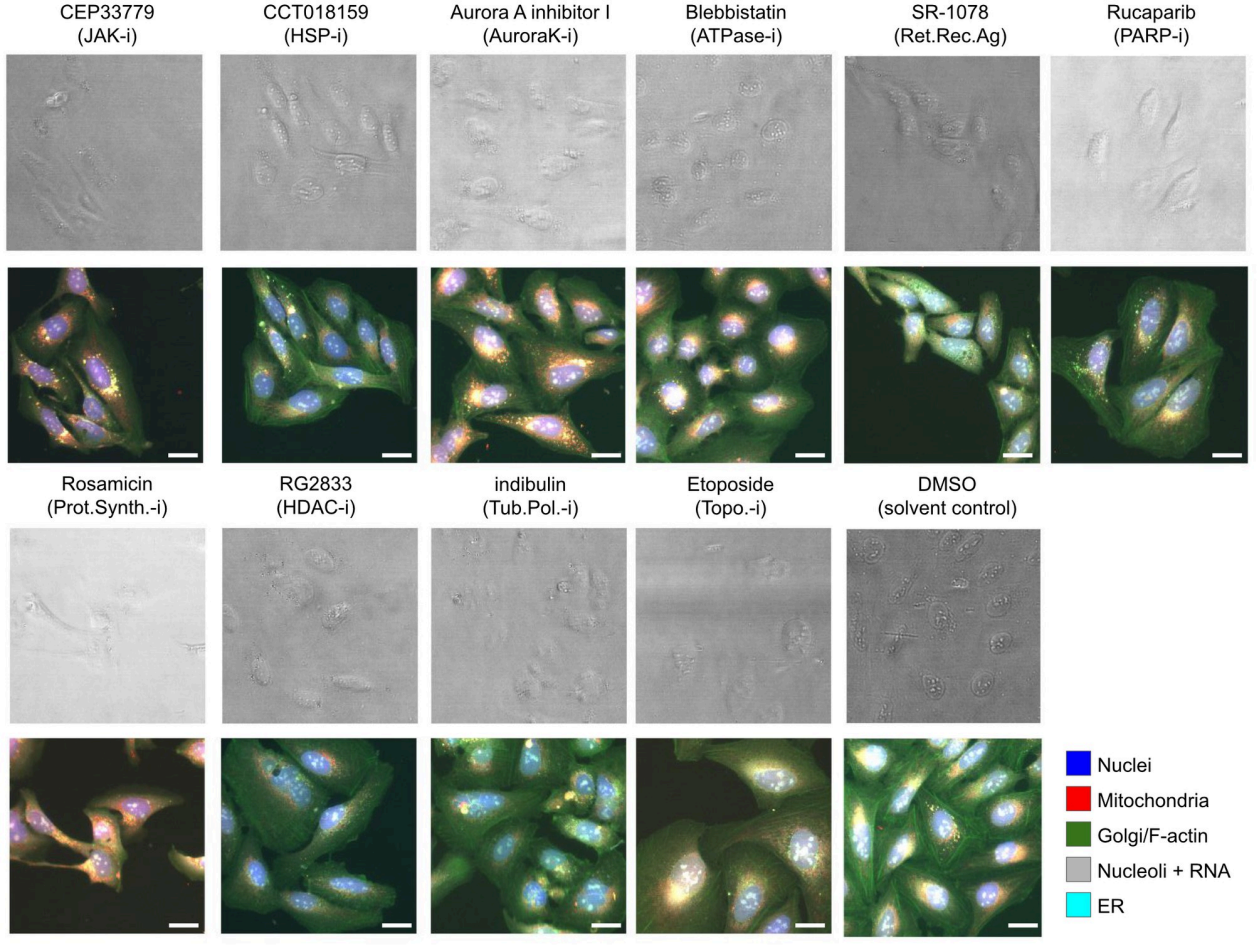
Examples of the image data used. Example BF and FL images for the ten MoA classes and the DMSO solvent control. The panel titles give the compound names for the selected images with the MoA abbreviation in parenthesis, where -i stands for inhibitor and Ag. for agonist. The top images in each case show an overlay of the six BF z-planes, and those below show merged images for the FL channels. The scale bars in the images represent 20 *μ*m.

In this study, we compared the performance of CNNs trained on Cell Painting fluorescence images (five channels) to those trained on brightfield images (six z-planes) for predicting ten MoA classes for U2OS cells treated with various compounds. As a reference/benchmark, we also trained neural networks on CellProfiler-derived features from the fluorescence images. Example Cell Painting images and their BF counterparts for the ten MoA classes and the DMSO are shown in Fig 1.

## Materials and methods

### Dataset

We used image data for compounds belonging to ten MoA classes (MoAs that we believed would be reasonably separable and that had a sufficient number of compounds (n) associated with them in our assay). The assay from which out data was taken included 865 compounds belonging to 225 MoA classes, however, for the majority of cases there were less than five compounds per MoA class. The 10 MoAs selected, with n = 231 compounds in total, were: ATPase inhibitors (ATPase-i, n = 18); Aurora kinase inhibitors (AuroraK-i, n = 20); HDAC inhibitors (HDAC-i, n = 33); HSP inhibitors (HSP-i, n = 24); JAK inhibitors (JAK-i, n = 21); PARP inhibitors (PARP-i, n = 21); protein synthesis inhibitors (Prot.Synth.-i, n = 23); retinoid receptor agonists (Ret.Rec.Ag, n = 19); topoisomerase inhibitors (Topo.-i, n = 32); and tubulin polymerization inhibitors (Tub.Pol.-i, n = 20). The compounds were administered at a dose of 10*μ*M to U2OS cells, and exposed for 48h, in 384 well plates. Each compound-level experiment was replicated six times with three technical replicates, i.e. compound treatments on the same plate, and two biological replicates, i.e. treatments on different plates. The compounds were distributed across eighteen microplates using PLAID (Plate Layouts using Artificial Intelligence Design, [15]), a constraint-programming-based method that aims to limit unwanted bias and batch effects, whereby the replicate compound treatments were placed at different well locations in the two biological replicate plates. Images (16-bit, 2160x2160 pixels) were captured with a 20X objective at five sites/fields-of-view in each well, with five fluorescence channels for the FL data and six evenly spaced z-planes for the BF data. See S1 Text for more details.

### Quality control

A common preprocessing strategy when working with FL data is to use automated methods to remove corrupted images, such as those with a high level of blurring or suffering from saturation artefacts [9]. These quality control (QC) metrics have been developed and implemented in CP for FL images [16]. In our study, we chose not to remove images based on this QC so as not to give the FL models an unfair advantage in our comparison. We note, however, that based on this QC, none of the FL images we used was flagged for saturation issues, although some blurred images were present. Given that a common data augmentation strategy when training CNNs is to purposefully blur the images, we do not expect their presence to have had an overly detrimental effect on the model predictions.

### Data splitting

We performed five splits of the data, at the compound level, into training, validation, and test sets. The splitting was performed in a stratified manner based on the proportion of compounds for each MoA. Each of the five test sets contained approximately 20% of the data, with no overlap of compounds. For each split, the remaining non-test data was shuffled at the compound level, and assigned to training or validation in a stratified manner, with 80% to training and 20% to validation. DMSO data was added to the sets with five, one, and two wells per plate for training, validation, and testing, respectively. These sets of DMSO wells were the same for each of the five splits. Overall, this corresponded for each split to ca. 4900 images for training, 1200 for validation and 1500 for testing, of which 400, 60 and 60, respectively, were DMSO.

### Normalization & background correction

For FL image data, a common normalization strategy is to use the mean and standard deviation of the pixel intensities of the control DMSO wells in each plate, to mitigate plate-level effects [9]. This normalization is performed separately for each imaging channel. For BF, there is no well-established normalization protocol. We, therefore, compared, for both imaging modalities, the effect of performing either DMSO plate-level normalization or site-level normalization (normalizing each image separately based on the mean and standard deviation of its pixel intensities). For the BF data, but not the FL data, the illumination across the images was very inhomogeneous, this background effect was corrected by first smoothing the images with a Gaussian filter of size 101x101 pixels to get the average background illumination in the image. This background image was then subtracted from the original image to remove the uneven illumination effects.

### Model training

For all the comparisons made we used a standard ResNet-50 [17] model with consistent hyper-parameters and training strategies. These multi-class classification models (predicting the class labels for 11 classes, i.e. the 10 MoAs and the DMSO control class) had 5 channel input images for the Cell Painting data, corresponding to the 5 FL channels, and 6 channel input images for the BF data, corresponding to the 6 z-planes. See S2 Text for more details.

### CellProfiler features

To benchmark the performance, we extracted CellProfiler (CP) features from the FL data on which we trained a fully-connected neural network with one hidden layer of 512 neurons followed by a RELU activation and batch normalization. We extracted 2009 features from the five-channel FL images, including those related to (in CP parlance) ‘AreaShape’, ‘Correlation’, ‘Granularity’, ‘Intensity’, ‘Neighbors’, and ‘RadialDistribution’. See S1 Text for more details on the CP feature extraction. The features were then mean aggregated to get a site-level profile of the features. The site-level features were then normalized at the plate level with the mean and standard deviation of the DMSO sites in the plate.

## Results

We first compared the impact of the different normalization techniques in the modalities with the CP baseline performance. Table 1(a) shows comparative test set macro-F1 scores for the five data splits for the models trained on BF and FL data. We report the macro-F1 score as this gives equal weight to each of the individual F1 scores and is thus not influenced by the imbalance in the amount of data for each MoA class. Table 1(b) shows the combined F1 scores (across all five tests sets, i.e. for all the compounds in our dataset) with respect to the ten MoA classes and the DMSO. The BF models perform competitively with respect to the FL models and the CP baseline when plate-level DMSO normalization was applied to the input images. When imaging site normalization was applied to the input data, the overall performance was lower, although comparable, for the two modalities. The difference in performance between the normalization techniques is similar in both modalities, however, the decline is slightly larger in FL models. S1 Fig show the confusion matrices for the BF and FL models.

**Table 1:** Comparison of the results for the models trained on BF and FL images and CP features. The subscript *dmso* is for the cases where DMSO plate-level normalization was applied to the data and the subscript *site* is for when the data was normalized at the imaging site level.

(a) Macro-F1 scores on the test sets for the five data splits.					
	BF_dmso_	FL_dmso_	BF_site_	FL_site_	CP
Split 1	0.738	**0.777**	0.662	0.661	0.771
Split 2	**0.821**	0.799	0.770	0.762	0.801
Split 3	0.724	**0.793**	0.654	0.677	0.718
Split 4	0.710	0.738	0.676	0.645	**0.739**
Split 5	0.728	0.716	0.708	0.688	**0.736**
(b) F1 scores per MoA across all five test sets.					
	BF_dmso_	FL_dmso_	BF_site_	FL_site_	CP
ATPase-i	0.605	0.701	0.650	0.683	**0.779**
AuroraK-i	0.683	0.675	0.713	0.671	**0.746**
HDAC-i	0.756	0.773	0.766	**0.785**	0.740
HSP-i	0.738	0.730	**0.756**	0.682	0.676
JAK-i	**0.675**	0.653	0.405	0.429	0.607
PARP-i	0.895	0.886	0.789	0.748	**0.912**
Prot.Synth.-i	0.711	**0.793**	0.520	0.646	0.711
Ret.Rec.Ag	0.740	0.769	0.767	**0.796**	0.786
Topo.-i	**0.780**	0.728	0.702	0.651	0.742
Tub.Pol.-i	**0.887**	0.854	0.850	0.865	0.845
DMSO	0.790	**0.866**	0.836	0.691	0.809
Macro average	0.751	**0.766**	0.705	0.695	0.759

### Compound-level accuracy analysis

**Fig 2:**
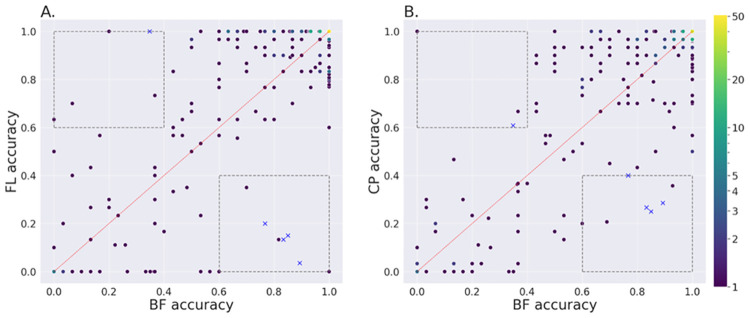
Compound-level accuracy for DMSO normalized input. Comparison of the accuracy at the compound level, across all five test sets, for the BF models with respect to FL models, and CP feature-based models. The input data was normalized based on the DMSO on each plate for all three cases. Each dark dot represents a compound. Brighter dots represent multiple compounds with the same accuracy score. **A.** BF against FL; **B.** BF against CP. In the boxes at the bottom right and top left, thresholded at accuracy values of 0.6 and 0.4, the compounds shown with blue crosses were consistently better for BF than both FL and CP or consistently worse, respectively.

To further explore the performance differences between the models, we compared the compound-level accuracies, as shown in Fig 2, for the models based on plate-wise DMSO normalization. The Pearson correlation coefficient between BF and FL was 0.744, whereas between BF and CP it was 0.798 which indicates that prediction errors made by the BF models are more correlated with the CP benchmark than with the FL. The correlation between FL and CP models was the highest, at 0.828, as expected since they are both based on FL images. Bottom-right and top-left sections of the plots (shown by the dashed boxes in Fig 2) are interesting regions as they highlight the compounds where the BF performance was better, or worse than the counterparts, respectively. We identified four compounds where the BF performance was consistently better and one compound where it was consistently worse than both the FL and CP models. The results overall indicate that there are multiple compounds in the dataset that exhibit morphological changes which can be picked up by the deep learning models applied to BF images. The compound in these interesting regions were different, although with some overlap, for the analyses based on imaging site normalisation (see S2 Fig). In this case there was one compound in the consistently better region, a compound also selected when using DMSO normalization, and seven compounds in the consistently worse region, one of which was also selected based on DMSO normalization.

### Biological relevance of activation maps

**Fig 3:**
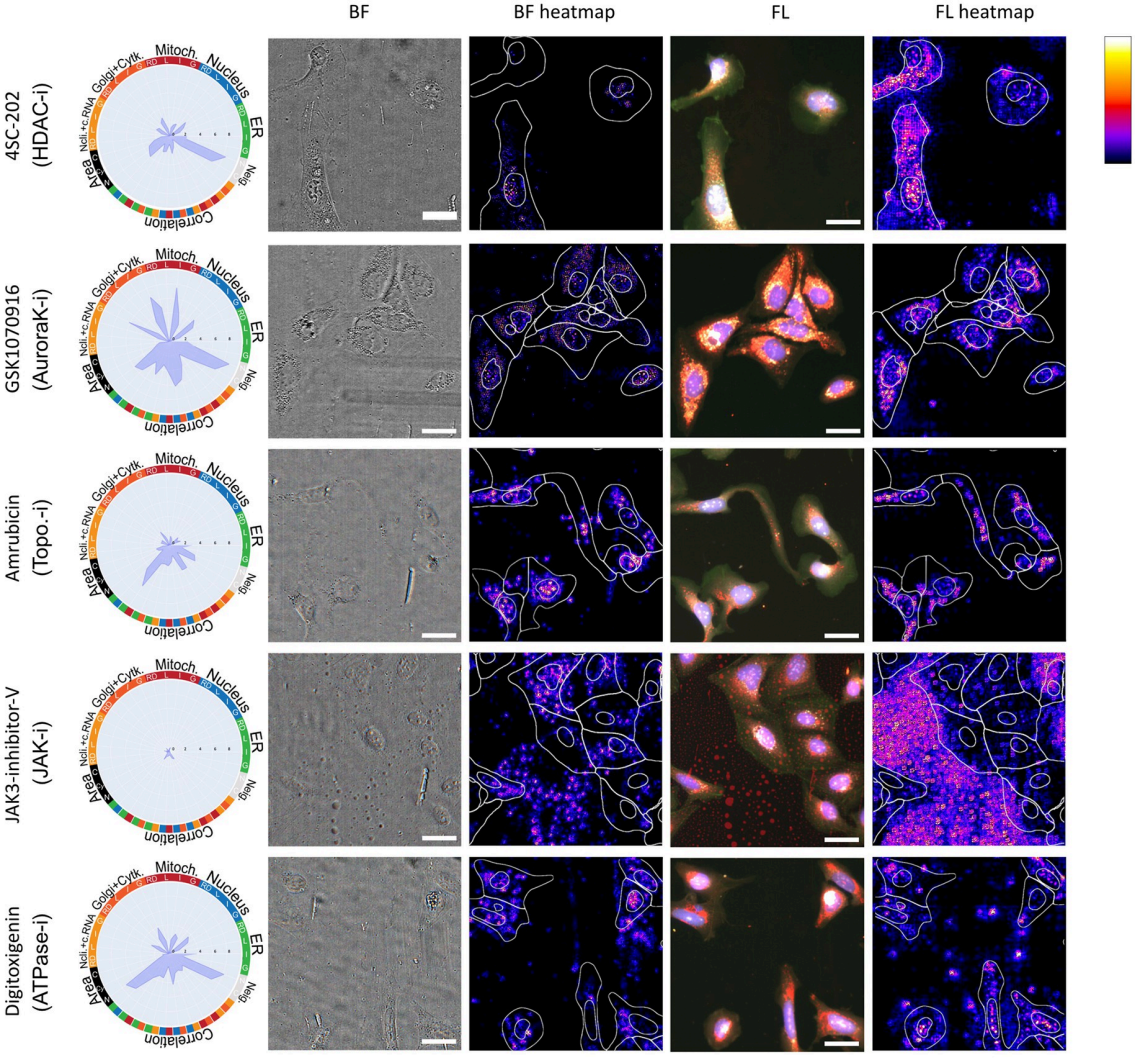
Explainable AI plots. Radar plots, BF and FL images and their corresponding activation heatmaps for four compounds that were considerably better predicted by BF than both FL and CP (first four rows), and one compound for which the opposite was true (last row), for the models based on DMSO normalization of the input images. The radar plots show the affected morphological features according to the CP data. A full description of the labels in the radar plots can be found in the CP feature extraction section of the supplementary material. The BF images show an RGB composite of the first 3 z-planes after illumination correction. The FL images show a merge of the five fluorescence channels with nuclei in blue, mitochondria in red, Golgi/F-actin in green, nucleoli and RNA in grey and ER in cyan. The scale bars in the images represent 20 *μ*m. In the heatmaps, the nuclei and cell boundaries, based on CellPose segmentation, are shown. The scale bar in the top right shows the relative importance of the areas highlighted in the heatmaps.

To further explore the compounds where the BF models performed significantly better or significantly worse than the FL and CP models, we used guided backpropagation [18] to determine which areas in the images were most activated by the models when making their class predictions. Fig 3 shows examples of the activation heatmaps for the four compounds better predicted by BF and one compound worse for the models based on DMSO normalization of the input images. The compound 4SC-202 (BF accuracy = 0.892, FL accuracy = 0.036, CP accuracy = 0.286) exhibits distinct activation patterns for the two modalities. In the FL heatmap, the focus is on the full cell body, whereas in the BF heatmap, there is a strong activation for the small vesicles that are visible in the BF images but that are not stained for in the Cell Painting protocol. In fact, many compounds, including HDAC inhibitors, affect lipid metabolism [19, 20]. For GSK1070916 (BF acc. = 0.859, FL acc. = 0.150, CP acc. = 0.250), there is an overlap in the areas activated for BF and FL. However, for the FL image, there is oversaturation in the signal (for the Golgi, ER and mitochondria), which could be hampering the predictive performance of the FL model. For Amrubicin (BF acc. = 0.833, FL acc. = 0.133, CP acc. = 0.267), the BF heatmap appears to focus on the nuclear areas and nucleoli, whereas the FL heatmap is more activated for perinuclear regions. Amrubicin is a topoisomerase inhibitor that affects the transcription of ribosomal DNA, which might result in nucleoli alterations. We hypothesise that changes in the nucleoli are highly specific for the MoA of this compound and can contribute to the high accuracy of the BF model [21]. For the compound JAK3-inhibitor-V (BF acc. = 0.767, FL acc. = 0.200, CP acc. = 0.400), we can observe undissolved compound, especially in the background of the image. The radar plot and grit score (0.886, see below) indicate that there is almost no morphological change induced by this compound, which is likely due to its low bioavailability. The FL model is particularly affected by the compound aggregates in the image, visible by the strong signal in the background areas. The BF heatmap seems to pick up the droplets specifically and thus may have an advantage over the FL model. A quality control step would have removed images that are corrupted by these types of artefacts, however, such removal was not used in this study so as not to give the FL models an advantage. Finally, for the compound Digitoxigenin (BF acc. = 0.348, FL acc. = 1.000, CP acc. = 0.608), where the BF model performed significantly worse than the FL and CP models, the heatmaps appear relatively similar. The FL model picks up on small aggregates in the mitochondria channel, a technical artifact that might have contributed to its good performance. The observations highlighted above are common across the other imaging sites and wells for these five compounds.

### Grit score analysis

The grit score [22] (See S1 Text for more details) indicates the phenotypical strength of the signals captured by the FL images, i.e. more compound-specific signals will be present in images with higher grit scores. Hence, to test if the learned models capture the phenotypic effects, one can compare the performance of the learned models with the grit score. If the learned features encode the phenotypical effects of the MoAs, the performance of the models should increase with the grit score. Furthermore, the behaviour of the models trained on different modalities and normalization can be compared against perturbation strengths to assess the difference in the learned features. For this comparison, all the images in the test sets were sorted by grit scores and binned into ten bins with an equal number of samples. The accuracy was calculated and reported with each bin’s mean grit score.

**Fig 4:**
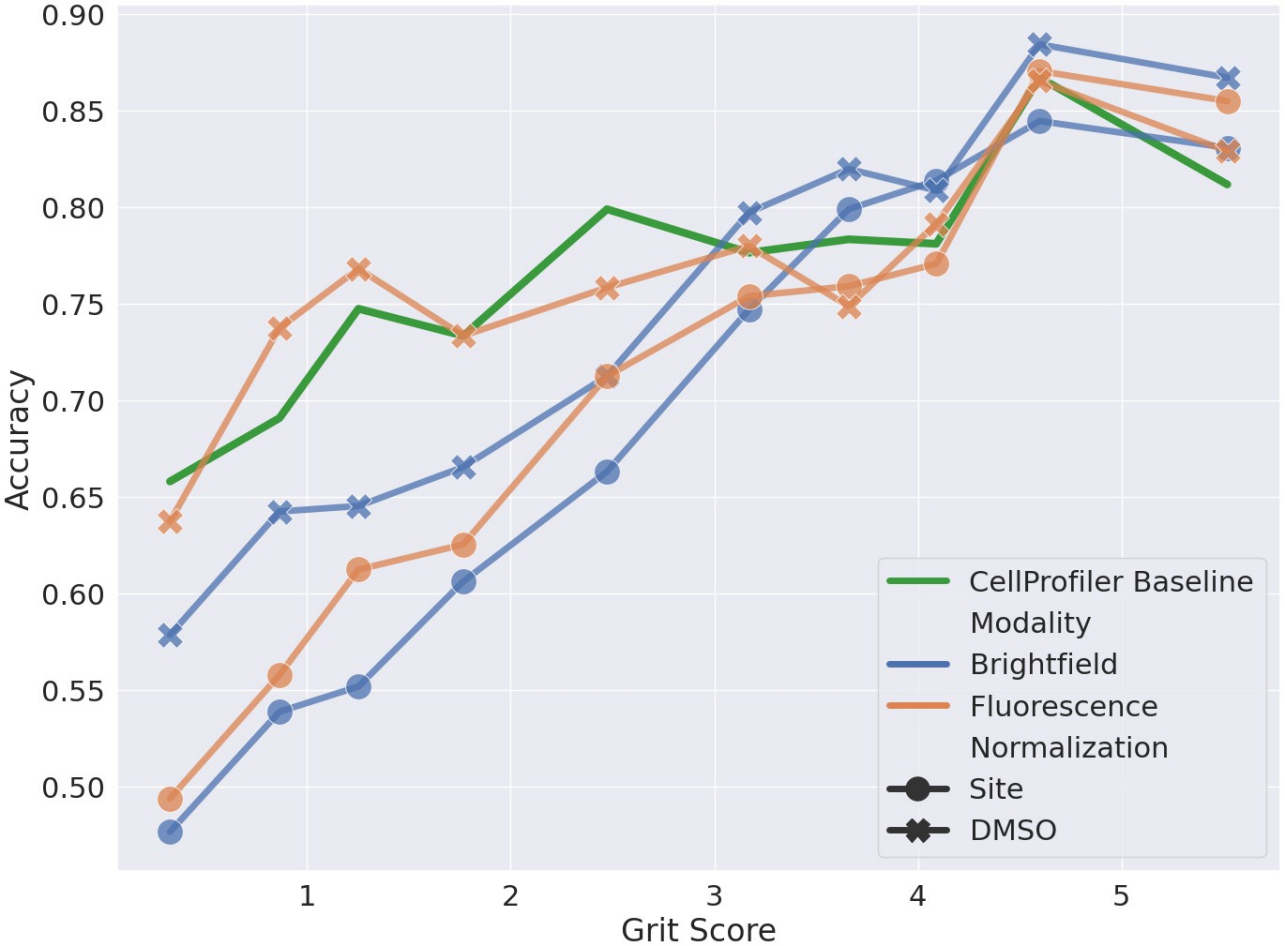
Grit score vs accuracy. Comparison of accuracy at different grit scores for BF (blue), FL (orange), and CP feature-based (green) models across all five test sets using either plate-based DMSO normalization or normalization at the imaging site level.

Fig 4 shows the accuracy vs the grit score of the BF and FL models with different normalization techniques compared to the models applied to the CP features. All the learned models show an upward trend in performance with increasing grit scores, indicating that the models do capture the phenotypical effects associated with the MoAs. However, the learned models perform worse than CP at a lower grit score, except for the FL DMSO normalized model. Models with site-level normalization perform worse than the DMSO-level normalization at a lower grit score for both modalities. This can be explained by the lack of relative intensity information in the site-normalized data as compared to the DMSO normalized data, where the relative intensity within a plate is preserved. When the phenotypical signal is low, the DMSO normalized models seem to utilise this information for the prediction task. As the signal strength increases, the difference in the performance of different normalization techniques decreases, indicating the capture of the phenotypical signal. The BF models seem to struggle at lower grit scores but catch up with the FL models at higher grit scores indicating that BF models perform comparable to the FL when a high phenotypical signal is present.

### Influence of technical variations

Our experimental design and image pre-processing aimed to eliminate unwanted technical variations, specifically through the use of PLAID and plate-based DMSO or site-based normalization, however, given that we only used a subset of the assay data for our analysis the compounds and MoAs were unevenly distributed across the 18 microwell plates (ranging from 1–4 for the latter and 1–67 for the former, see S3 Fig). The DMSO class was the only class present in all 18 plates. Unwanted/nuisance technical variations can arise from differences in lab conditions, microscope calibration, and day-to-day fluctuations in humidity and temperature [9, 23, 24]. While these “batch effects” tend to be larger when data from multiple laboratories is combined, they can still occur between the imaging plates collected in a single lab. Although subtle to the eye, if these batch effects are correlated with the biological signal, then neural networks may leverage them and overfit to the data [25].

**Fig 5:**
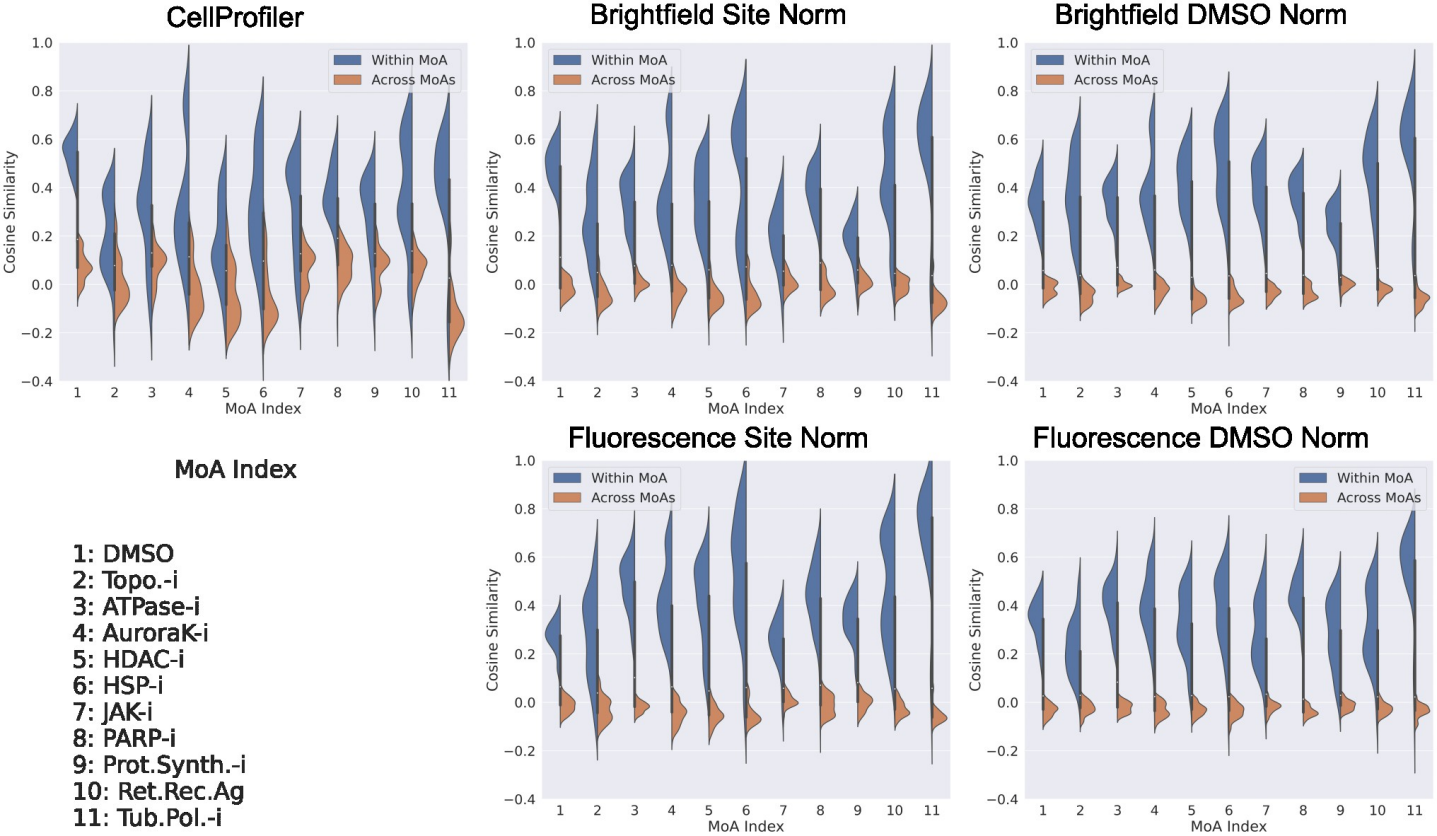
Cosine similarity within and across the MoA classes. Cosine similarity within (blue) and across (orange) the MoA classes for the test set CP features and for features extracted from the test data for the BF and FL models based on the two different normalization (norm) strategies.

**Fig 6:**
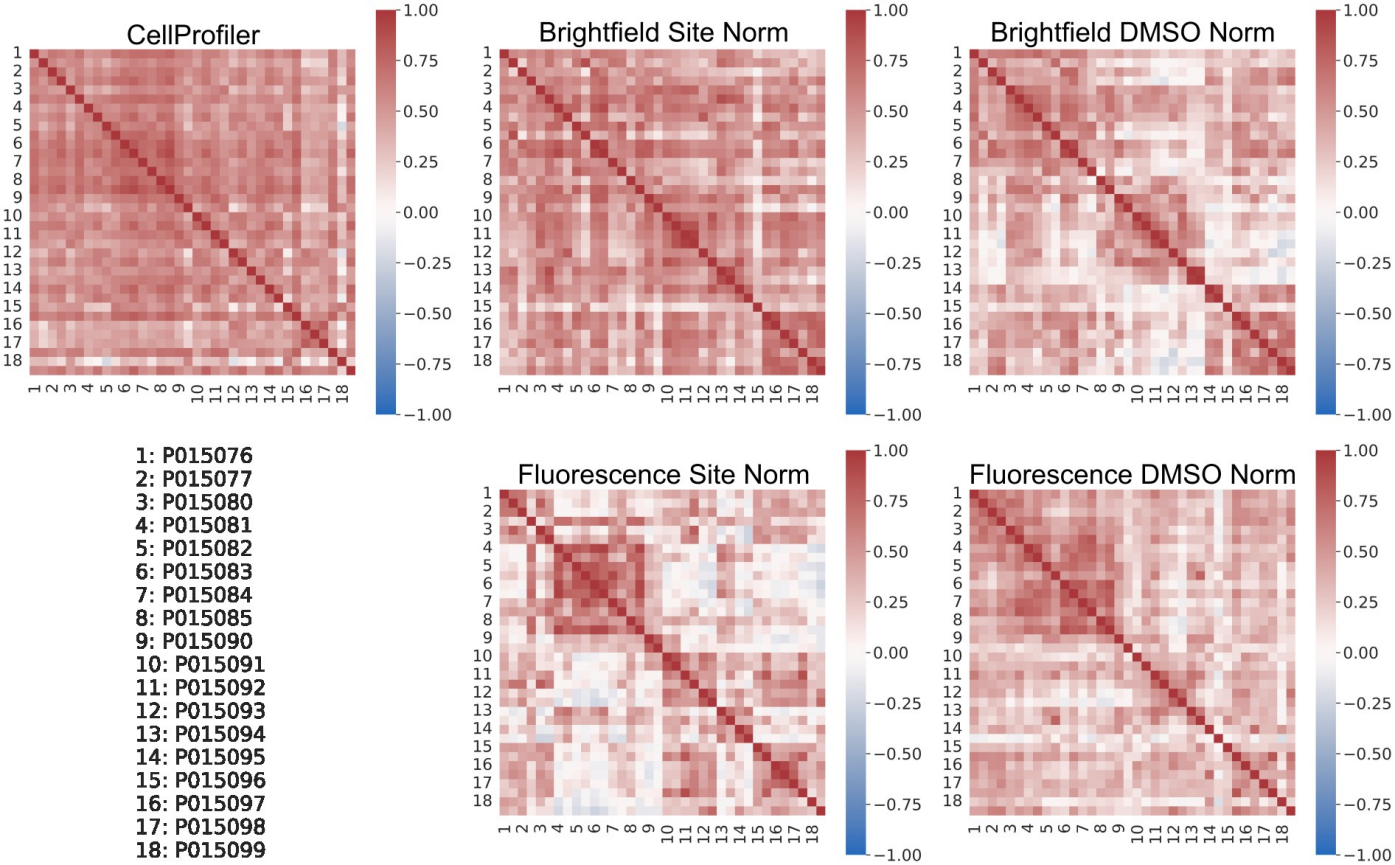
Pearson correlations for the features between the imaging plates. Pearson correlations for the features extracted from the test set DMSO wells (averaged across the five splits) between the 18 imaging plates used. Note that there were two DMSO test wells for each plate.

We used features extracted from the trained models for the test data to investigate if any unwanted technical variations were present. The BF and FL features were extracted from the penultimate layer of the ResNet50 model, the vector of length 2048, i.e. the 2D adaptive average pooling layer prior to the final fully connected output prediction layer. From Fig 5 it can be seen that these extracted features for both BF and FL can clearly distinguish each MoA class from the others, for both of the normalization strategies, and with a larger separation than that obtained from the CP features. However, based on the extracted features from the test set DMSO data, there is evidence of plate effects (Fig 6). In an ideal case there should be a reasonable amount of correlation between all the plates and no systematic patterns for this control data. This was the case for the CP features and to some extent for the features extracted from the BF models with site normalization applied to the input data. For the other cases, however, there was more variability in the correlation between the plates. For the features extracted from the FL models with site normalization of the input data, in contrast to the case for BF, there were more pronounced plate effects than when DMSO normalization was applied.

**Fig 7:**
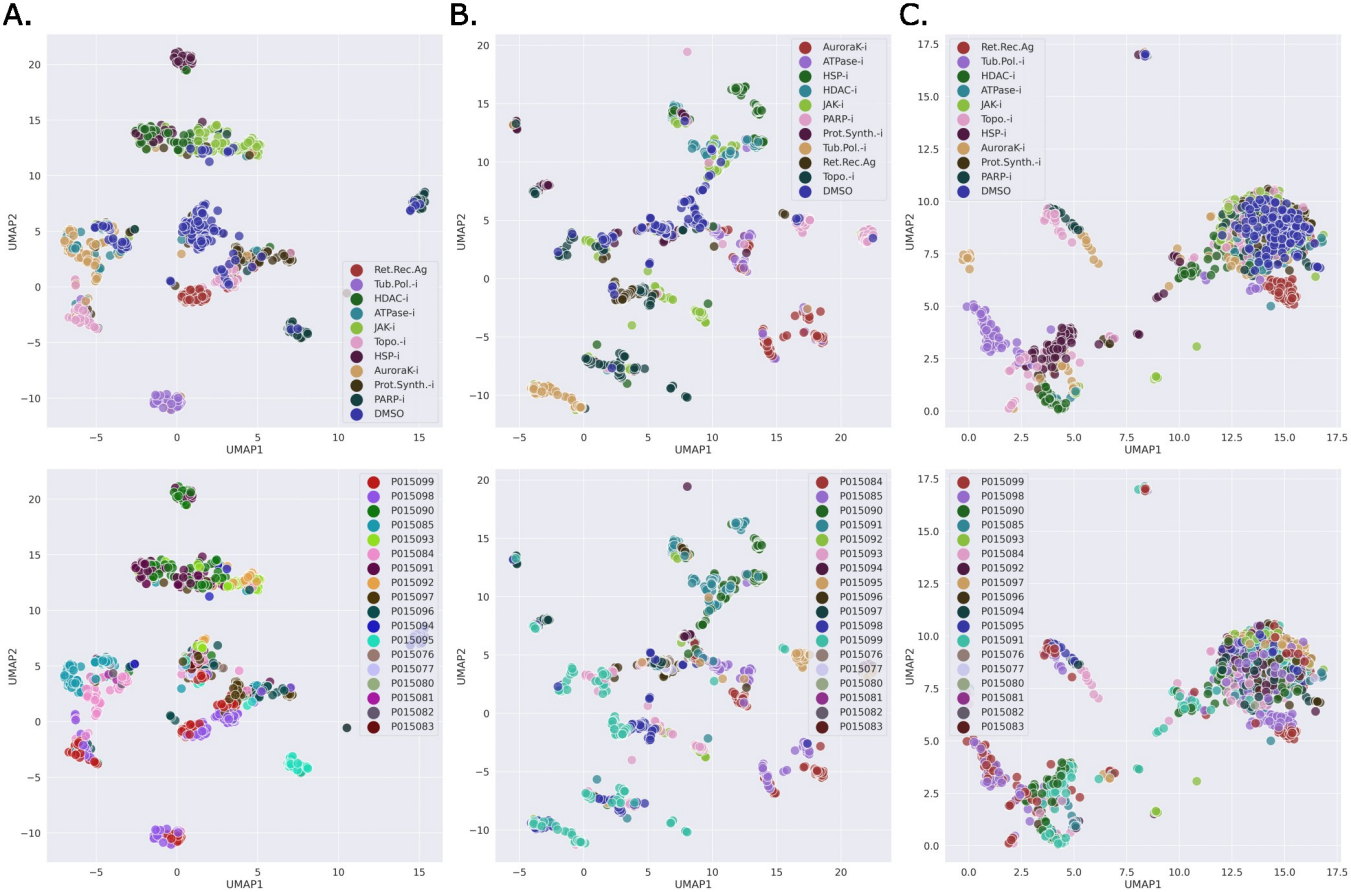
UMAP projections (DMSO normalization). UMAP plots of features learned by the BF and FL models (based on DMSO normalization of the input images), and the raw CP features of the test set for the best-performing split (split 2), colour-coded by MoA (top-row) and by plate (bottom-row). The UMAP was fitted on the training data of the split and the test set features were transformed. **A.** BF features; **B**. FL features; **C**. CP cell-based features.

Fig 7 shows the UMAP [26] projections for the extracted test set features fitted on the train set features with respect to the MoAs and the plates for the DMSO normalized models. A similar plot for the site-level normalization is shown in S4 Fig. No obvious biases could be observed from these plots. However, S5 Fig, showing UMAP projections colour coded by compounds and shape coded by plate, revealed cases where the bias was present at the MoA level. For HDAC-i, no plate bias was observed for BF and FL. For BF, the plate bias can be observed in the PARP-i where the same compounds (CBK278031, CBK309255) in different plates are clustered separately. Similar bias can be observed in FL, however, to a lesser extent. For Topo-i, the two clusters can be observed in BF with different compounds in each indicating no plate-level bias. For FL, slight plate bias can be observed for CBK201030 and CBK289760 where the points from different plates are farther apart than the points within the plate for the compounds. From the UMAP plots for CP (column C.), there was no evidence of plate-level bias. This is perhaps due to the fact that the features were extracted from the segmented cells, as opposed to from the entire images, as they were for the BF and FL models. S6 Fig shows similar plots for site-level normalization for the three MoAs.

### Downstream analysis

Moshkov et al. [23], using a weakly supervised deep learning approach for extracting features from Cell Painting image data, also found that their learned representations encoded both phenotypically relevant features and confounding factors. To further analyse the feature representation and the utility of the features with respect to new data, we used the features to perform a downstream biological matching task similar to [23]. We used our trained models to extract features for a new set of MoAs (those with at least ten compounds) from the plates remaining in the original imaging assay, i.e. those not used in the original dataset. This gave us six new MoAs distributed over four plates: CC Chemokine Receptor Antagonist (CCR.Ag); DNA Polymerase Inhibitor (DNAP-i); DNA Synthesis Inhibitor (DNAS-i); EGFR Inhibitor (EGFR-i); Cyclooxygenase Inhibitor (COX-i); and Glucocorticoid Receptor Agonist (GR.Ag). See S7 Fig for the number of compounds per MoA across the new plates. The features for all the sites were extracted and aggregated by mean to form well-level profiles. The performance on a biological matching task was evaluated by computing mean average precision (mAP) over all the wells. For each reference well, the cosine similarity of all the other query wells is calculated and sorted in decreasing order into a list. A query well in the list is considered a hit if the MoA of the query well matches the reference. The precision and recall at each query in the list are computed until all the wells in the MoA are found. The average precision is calculated for every well in the dataset with the rest of the wells as the query, and the mAP is reported.

Under the assumption that a large variation found in control DMSO samples between plates/batches is associated with these confounding factors, [23] proposed a linear sphering transformation to reduce the variation of the confounding factors and amplify the phenotypically relevant features. This transformation aims to reduce the features of the control wells to a white noise distribution and to correct the treated wells accordingly. The amount of sphering to apply is controlled by a regularization parameter, with smaller values corresponding to a higher degree of sphering. As we saw the evidence of the plate effect in the previous section, we opted to use the transformation for the biological matching task and report the results.

**Fig 8:**
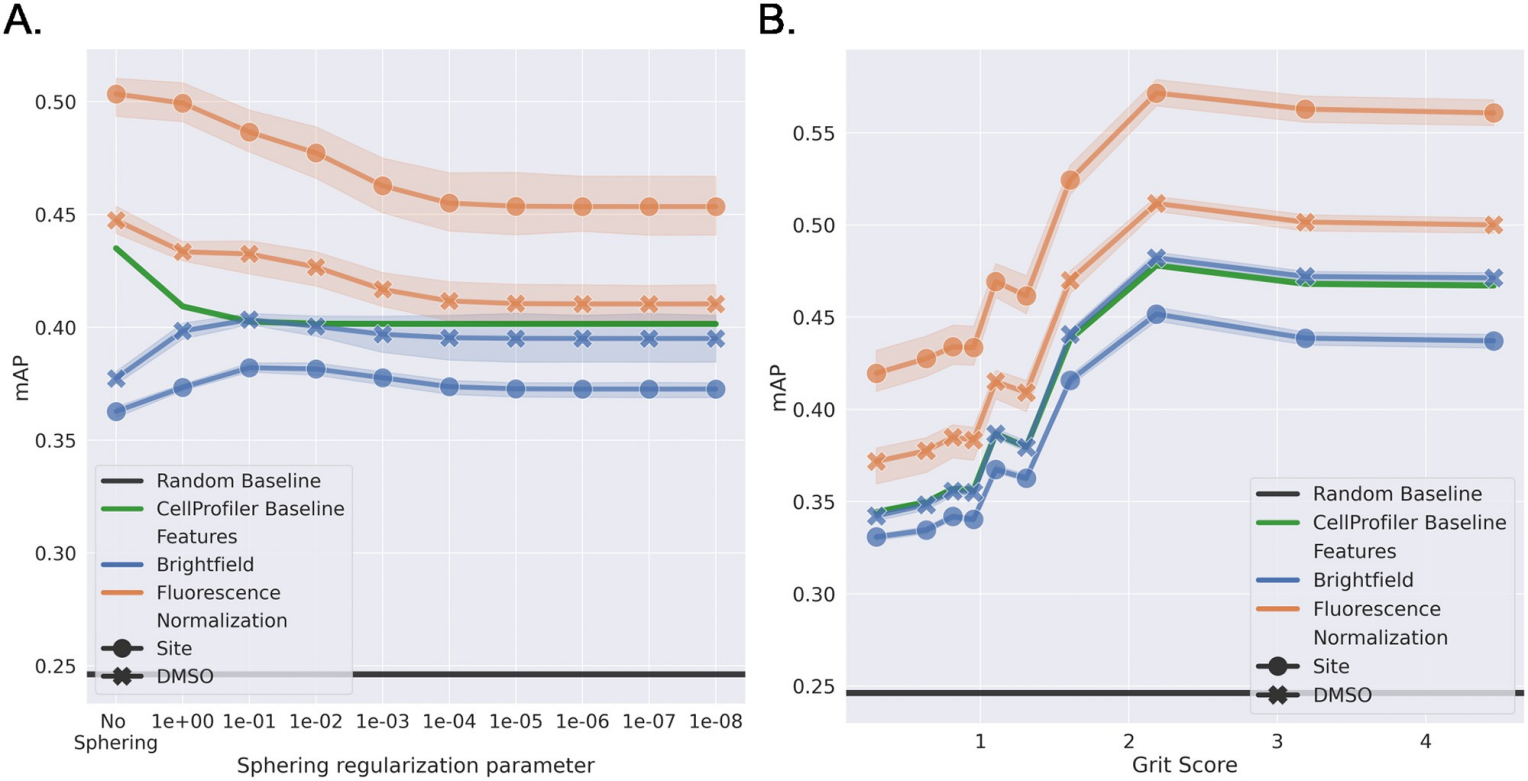
Downstream task. **A.** The influence of applying different levels of the sphering transformation on the mean average precision (mAP) for the biological matching downstream task. **B.** Comparison of the mAP at different grit scores with the sphering regularization parameter of 0.1. The panels show BF (blue), FL (orange), and CP feature-based (green) using either plate-based DMSO normalization or normalization at the imaging site level.

Fig 8A shows the mAP of the different models under increasing regularization. FL models outperform BF models and the CP baseline at every regularization strength. This indicates that features the FL models learn are better than BF features for the downstream task. Surprisingly, the site-normalized models in FL performed better than the DMSO-normalized models. The performance of the BF models is close to that of the CP baseline and higher than the random performance. This would suggest that the BF models are encoding biologically relevant features, and the generalization performance of the models matches the untrained features extracted from the CellProfiler pipeline. Fig 8B shows the mAP performance with the grit score at the regularization parameter of 0.1. This was chosen as the BF model performed best at this value, however, a similar trend was observed for other parameter values. Here, we observe a similar trend as seen in Fig 4 with performance increasing with the grit score, suggesting that CNN models can even distinguish different strong phenotypical signals not present in the training set.

### Reproducibility and alternative architectures

To confirm the reproducibility of our results, we trained our models three times with different seeds (see S1 and S2 Tables for the results with DMSO-level and site-level normalization, respectively). For the five splits of the test data and for the ten MOAs and the DMSO class, there was low variability for the differently seeded runs of the models, further corroborating our results given above. To assess the impact of the neural network choice on our analysis, we compared the results obtained using the ResNet-50 architecture to those obtained using two alternative architectures (DenseNet-169 [27] and EfficientNet-B3 [28]). From S3 and S4 Tables, showing the results based on DMSO and site normalization respectively, the best overall results for BF were achived with ResNet-50 and for FL with EfficientNet-B3. Note that for the BF DMSO normalization EfficientNet-B3 models three of the splits failed to converge, hence the relatively poor performance in this case.

## Discussion

In our work, we found comparable predictive performance for models based on BF images to those based on both FL images and CP features derived from them. We found that the BF models made similar prediction errors to both the FL and CP models. This shows that the features required to delineate the MoAs are present in the BF images and can be extracted by deep learning models.

Other studies prior to ours have demonstrated the potential utility of BF image data. For instance, [29] were able to discriminate single-gene mutant cells from wild-type cells and further found that functionally close genes shared similar BF-based feature profiles. For flow cytometry, [30] successfully discriminated multiple cell lines and [31] achieved high accuracy for distinguishing between drug-treated and -untreated cells, an accuracy that increased with dosage and treatment time. For 3D cancer sphere cultures, using CNNs trained on BF data, [32] reliably estimated drug efficacy for three chemotherapy drugs. For BF time-lapse data, [33] developed a method for exploring variations in cell behaviour in response to drug treatments, which they exemplified using drugs that modify cytoskeletal protein dynamics.

In our work, the models trained on the CP features had lower predictive performance than CNNs trained on the FL images, when using ResNet-50 and DMSO-based normalization of the input images. A previous study by [34] found this also to be the case for predicting drug activity across multiple Cell Painting image assays. We emphasize here, however, that our purpose was not to train the best possible predictive model for either the BF or FL image data or the CP feature data, but rather to use a well-established CNN network, ResNet-50, to compare the BF and FL modalities and to use the CP model as a benchmark. In our final investigation, comparing three alternative CNN architectures, we found that ResNet-50 gave the best performance for BF and EfficientNet-B3 gave the best performance for FL.

In our analysis we uncovered some plate level bias in our results, in spite of the fact that we used PLAID and plate based DMSO normalization or imaging site normalization. Performing normalization of the input data at the image level removed some of this bias for the BF models but not for the FL models. This somewhat extreme form of normalization likely hampered the overall predictive performance of the models as it will have removed much of the relative intensity differences between the compound treatments. However, as a means of eliminating some of the unwanted technical biases, image based normalization was also used by [23] in their weakly supervised deep learning approach for extracting features from Cell Painting image data. The primary reason that our models were able to exploit plate information is that the data used was taken from an assay not specifically designed for our study and unfortunately the compounds and MoAs were very unevenly distributed among the plates. An experimental design with a more even distribution of the compounds and MoAs across the plates would eliminate such plate level biases and allow retaining more of the relative intensity information.

We attempted to remove this plate-level bias by applying a linear sphering transformation to the features extracted from the images for a downstream biological matching task. Contrary to Moshkov et al. [23], however, we only found slight improvements in this biological matching for the BF data and drops in performance for both the FL and CP data. It is possible that nonlinear transformations would perform better. In spite of potential biasing effect of these confounding factors, methods for batch correction are somewhat under developed for microscopy imaging based analyses relative to other data modalities, e.g. gene expression data [23]. In our study these confounding factors were likely similar for both BF and FL as the images for both were taken from the same plates, and although we could not entirely eliminate these factors, the overall predictive performance was very comparable between the two imaging modalities. Our grit based analyses and explainable AI explorations also highlight that the models are learning important biological information, despite these confounding factors. A recently proposed method named “batch effects normalization” [25], provides knowledge of the batches or plates to the CNNs during training to potentially remove these confounding factors. Due to time limitations we did not explore this approach, but leave it as an avenue for future work.

Had the predictive performance been significantly poorer for the BF models than the FL models one avenue for exploration would have been to perform virtual staining [35–37] to generate virtually stained images from which to subsequently base the MoA prediction. However, going via this route would lose any morphological information that is only present in the BF images. We had several cases of compounds that were considerably better predicted by the BF model, relative to the FL models, suggesting that there are cellular compartments/organelles potentially picked up in the BF images that are not stained for in the Cell Painting protocol (such as lysosomes, endosomes and peroxisomes). For cases where the opposite was true (i.e. where the FL models performed well but the BF models did not) we hypothesize that this may be a result of cellular compartments stained for in the Cell Painting protocol, which were useful for prediction, but which are barely visible or not accessible in the BF images (such as the f-actin cytoskeleton).

The fact that deep learning can be used on BF images for MoA prediction holds great promise for live cell time-lapse studies, for which using FL data is problematic. With trained BF models we can track the cell populations over time to explore how the features evolve towards the MoAs after drug administration and how quick the process is. A wealth of interesting information will likely come from simply visualizing these temporal dynamics.

## Supporting information

S1 Text Data acquisition. Includes information on the cell culture, compounds, cell painting, image acquisition, CellProfiler feature extraction and the grit scores.
(PDF)

S2 Text Model training. Additional information on the model training strategy (optimizers, learning rate and augmentations applied).
(PDF)

S1 Fig Confusion matrices. Normalized confusion matrices, across all five test sets, when DMSO plate-level normalization was applied to the data (**A.** BF models; **B.** FL models) and when the data was normalized at the imaging site level (**C.** BF models; **D.** FL models). Labels are **0**:ATPase-i, **1**:AuroraK-i, **2**:HDAC-i, **3**:HSP-i, **4**:JAK-i, **5**:PARP-i, **6**:Prot.Synth.-i, **7**:Ret.Rec.Ag, **8**:Topo.-i, **9**:Tub.Pol.-i, **10**:DMSO.
(PNG)

S2 Fig Compound-level accuracy for site normalized input. Comparison of the accuracy at the compound level, across all five test sets, for the BF models with respect to FL models, and CP feature-based models. The input data was normalized separately for each imaging site for BF and FL and was normalized based on the DMSO on each plate for CP. Each dark dot represents a compound. Brighter dots represent multiple compounds with the same accuracy score. **A.** BF against FL; **B.** BF against CP. In the boxes at the bottom right and top left, thresholded at accuracy values of 0.6 and 0.4, the compounds shown with blue crosses were consistently better for BF than both FL and CP or consistently worse, respectively.
(PNG)

S3 Fig Distribution of compounds per MoA across the imaging plates. The number of compounds per MoA across each of the imaging plates used in our study. Note that there are pairs of plates, biological replicates, with the same compound treatments, but the treatments were located in different wells within each of the replicate plates.
(PNG)

S4 Fig UMAP projections (site-level normalization). UMAP plots of features learned by the BF and FL models (based on site-level normalization of the input images), and the raw CP features of the test set for the best-performing split (split 2), colour-coded by MoA (top-row) and by plate (bottom-row). The UMAP was fitted on the training data of the split and the test set features were transformed. **A.** BF features; **B**. FL features; **C**. CP cell-based features.
(PNG)

S5 Fig UMAP projections (DMSO normalisation, 3 MoAs). UMAP plots of features learned by the BF and FL models (based on DMSO normalization of the input images), and the raw CP features of the test set for the best-performing split (split 2) for three of the MoAs, color coded by compound and shape coded by plate. **A.** BF features; **B**. FL features; **C**. CP cell-based features.
(PNG)

S6 Fig UMAP projections (site-level normalisation, 3 MoAs). UMAP plots of features learned by the BF and FL models (based on site-level normalization of the input images), and the raw CP features of the test set for the best-performing split (split 2) for three of the MoAs, color coded by compound and shape coded by plate. **A.** BF features; **B**. FL features; **C**. CP cell-based features.
(PNG)

S7 Fig Distribution of compounds per MoA across the imaging plates for the downstream task. The number of compounds per MoA across each of the imaging plates used in our downstream analysis. Note that there are pairs of plates, biological replicates, with the same compound treatments, but the treatments were located in different wells within each of the replicate plates.
(PNG)

S1 Table Reproducibility (DMSO normalization). Comparison of the results for three model runs with different seeds when DMSO plate-level normalization was applied to the data.
(PDF)

S2 Table Reproducibility (site normalization). Comparison of the results for three model runs with different seeds when site-level normalization was applied to the data.
(PDF)

S3 Table Alternative architectures (DMSO normalization). Comparison of the test set F1 scores for three different architectures trained on BF and FL when DMSO plate-level normalization was applied to the data.
(PDF)

S4 Table Alternative architectures (site normalization). Comparison of the test set F1 scores for three different architectures trained on BF and FL when site-level normalization was applied to the data.
(PDF)

## References

[1] Trapotsi MA, Hosseini-Gerami L, Bender A. Computational analyses of mechanism of action (MoA): data, methods and integration. RSC Chemical Biology. 2022;3(2):170–200. doi: 10.1039/d1cb00069a 35360890 PMC8827085

[2] Ziegler S, Sievers S, Waldmann H. Morphological profiling of small molecules. Cell Chemical Biology. 2021;28(3):300–319. doi: 10.1016/j.chembiol.2021.02.012 33740434

[3] Caicedo JC, Singh S, Carpenter AE. Applications in image-based profiling of perturbations. Current opinion in biotechnology. 2016;39:134–142. doi: 10.1016/j.copbio.2016.04.003 27089218

[4] Thorn K. A quick guide to light microscopy in cell biology. Molecular Biology of the Cell. 2016;27(2):219–222. doi: 10.1091/mbc.E15-02-0088 26768859 PMC4713126

[5] Bray MA, Singh S, Han H, Davis CT, Borgeson B, Hartland C, et al. Cell Painting, a high-content image-based assay for morphological profiling using multiplexed fluorescent dyes. Nature protocols. 2016;11(9):1757–1774. doi: 10.1038/nprot.2016.105 27560178 PMC5223290

[6] Purschke M, Rubio N, Held KD, Redmond RW. Phototoxicity of Hoechst 33342 in time-lapse fluorescence microscopy. Photochemical & Photobiological Sciences: Official Journal of the European Photochemistry Association and the European Society for Photobiology. 2010;9(12):1634–1639. doi: 10.1039/c0pp00234h 20931137

[7] Thorley JA, Pike J, Rappoport JZ. Chapter 14—Super-resolution Microscopy: A Comparison of Commercially Available Options. In: Cornea A, Conn PM, editors. Fluorescence Microscopy. Boston: Academic Press; 2014. p. 199–212. Available from: https://www.sciencedirect.com/science/article/pii/B9780124095137000142.

[8] Carpenter AE, Jones TR, Lamprecht MR, Clarke C, Kang IH, Friman O, et al. CellProfiler: image analysis software for identifying and quantifying cell phenotypes. Genome Biology. 2006;7(10):R100. doi: 10.1186/gb-2006-7-10-r100 17076895 PMC1794559

[9] Caicedo JC, Cooper S, Heigwer F, Warchal S, Qiu P, Molnar C, et al. Data-analysis strategies for image-based cell profiling. Nature Methods. 2017;14(9):849–863. doi: 10.1038/nmeth.4397 28858338 PMC6871000

[10] Bougen-Zhukov N, Loh SY, Lee HK, Loo LH. Large-scale image-based screening and profiling of cellular phenotypes. Cytometry Part A. 2017;91(2):115–125. doi: 10.1002/cyto.a.22909 27434125

[11] Rohban MH, Singh S, Wu X, Berthet JB, Bray MA, Shrestha Y, et al. Systematic morphological profiling of human gene and allele function via Cell Painting. eLife. 2017;6:e24060. doi: 10.7554/eLife.24060 28315521 PMC5386591

[12] LeCun Y, Bengio Y, Hinton G. Deep learning. Nature. 2015;521(7553):436–444. doi: 10.1038/nature14539 26017442

[13] Gupta A, Harrison PJ, Wieslander H, Pielawski N, Kartasalo K, Partel G, et al. Deep Learning in Image Cytometry: A Review. Cytometry Part A. 2019;95(4):366–380. doi: 10.1002/cyto.a.23701 30565841 PMC6590257

[14] Kensert A, Harrison PJ, Spjuth O. Transfer learning with deep convolutional neural networks for classifying cellular morphological changes. SLAS Discovery: Advancing Life Sciences R&D. 2019;24(4):466–475. doi: 10.1177/2472555218818756 30641024 PMC6484664

[15] Francisco Rodríguez MA, Carreras Puigvert J, Spjuth O. Designing microplate layouts using artificial intelligence. Artificial Intelligence in the Life Sciences. 2023; p. 100073. doi: 10.1016/j.ailsci.2023.100073

[16] Bray MA, Fraser AN, Hasaka TP, Carpenter AE. Workflow and Metrics for Image Quality Control in Large-Scale High-Content Screens. Journal of Biomolecular Screening. 2012;17(2):266–274. doi: 10.1177/1087057111420292 21956170 PMC3593271

[17] He K, Zhang X, Ren S, Sun J. Deep residual learning for image recognition. In: Proceedings of the IEEE conference on computer vision and pattern recognition; 2016. p. 770–778.

[18] Springenberg JT, Dosovitskiy A, Brox T, Riedmiller M. Striving for Simplicity: The All Convolutional Net; 2015. Available from: http://arxiv.org/abs/1412.6806.

[19] Sun Z, Miller RA, Patel RT, Chen J, Dhir R, Wang H, et al. Hepatic Hdac3 promotes gluconeogenesis by repressing lipid synthesis and sequestration. Nature Medicine. 2012;18(6):934–942. doi: 10.1038/nm.2744 22561686 PMC3411870

[20] Chittur SV, Sangster-Guity N, McCormick PJ. Histone deacetylase inhibitors: A new mode for inhibition of cholesterol metabolism. BMC Genomics. 2008;9(1):507. doi: 10.1186/1471-2164-9-507 18959802 PMC2613157

[21] Lindström MS, Jurada D, Bursac S, Orsolic I, Bartek J, Volarevic S. Nucleolus as an emerging hub in maintenance of genome stability and cancer pathogenesis. Oncogene. 2018;37(18):2351–2366. doi: 10.1038/s41388-017-0121-z 29429989 PMC5931986

[22] Trapotsi MA, Mouchet E, Williams G, Monteverde T, Juhani K, Turkki R, et al. Cell Morphological Profiling Enables High-Throughput Screening for PROteolysis TArgeting Chimera (PROTAC) Phenotypic Signature. ACS Chemical Biology. 2022;17(7):1733–1744. doi: 10.1021/acschembio.2c00076 35793809 PMC9295119

[23] Moshkov N, Bornholdt M, Benoit S, Smith M, McQuin C, Goodman A, et al.. Learning representations for image-based profiling of perturbations; 2022. Available from: https://www.biorxiv.org/content/10.1101/2022.08.12.503783v2. 10.1038/s41467-024-45999-1 PMC10881515 38383513

[24] Ando DM, McLean CY, Berndl M. Improving Phenotypic Measurements in High-Content Imaging Screens; 2017. Available from: https://www.biorxiv.org/content/10.1101/161422v1.

[25] Lin A, Lu AX. Incorporating knowledge of plates in batch normalization improves generalization of deep learning for microscopy images; 2022. Available from: https://www.biorxiv.org/content/10.1101/2022.10.14.512286v1.

[26] McInnes L, Healy J, Melville J. UMAP: Uniform Manifold Approximation and Projection for Dimension Reduction; 2020. Available from: http://arxiv.org/abs/1802.03426.

[27] Huang G, Liu Z, van der Maaten L, Weinberger KQ. Densely Connected Convolutional Networks; 2018. Available from: http://arxiv.org/abs/1608.06993.

[28] Tan M, Le Q. EfficientNet: Rethinking Model Scaling for Convolutional Neural Networks. In: Proceedings of the 36th International Conference on Machine Learning. PMLR; 2019. p. 6105–6114. Available from: https://proceedings.mlr.press/v97/tan19a.html.

[29] Suzuki G, Saito Y, Seki M, Evans-Yamamoto D, Negishi M, Kakoi K, et al. Machine learning approach for discrimination of genotypes based on bright-field cellular images. npj Systems Biology and Applications. 2021;7(1):1–8. doi: 10.1038/s41540-021-00190-w 34290253 PMC8295336

[30] Meng N, Lam EY, Tsia KK, So HKH. Large-Scale Multi-Class Image-Based Cell Classification With Deep Learning. IEEE Journal of Biomedical and Health Informatics. 2019;23(5):2091–2098. doi: 10.1109/JBHI.2018.2878878 30387753

[31] Kobayashi H, Lei C, Wu Y, Mao A, Jiang Y, Guo B, et al. Label-free detection of cellular drug responses by high-throughput bright-field imaging and machine learning. Scientific Reports. 2017;7(1):12454. doi: 10.1038/s41598-017-12378-4 28963483 PMC5622112

[32] Zhang Z, Chen L, Wang Y, Zhang T, Chen YC, Yoon E. Label-Free Estimation of Therapeutic Efficacy on 3D Cancer Spheres Using Convolutional Neural Network Image Analysis. Analytical Chemistry. 2019;91(21):14093–14100. doi: 10.1021/acs.analchem.9b03896 31601098 PMC13134704

[33] Baar S, Kuragano M, Tokuraku K, Watanabe S. Towards a comprehensive approach for characterizing cell activity in bright-field microscopic images. Scientific Reports. 2022;12(1):16884. doi: 10.1038/s41598-022-20598-6 36207347 PMC9546915

[34] Hofmarcher M, Rumetshofer E, Clevert DA, Hochreiter S, Klambauer G. Accurate Prediction of Biological Assays with High-Throughput Microscopy Images and Convolutional Networks. Journal of Chemical Information and Modeling. 2019;59(3):1163–1171. doi: 10.1021/acs.jcim.8b00670 30840449

[35] Christiansen EM, Yang SJ, Ando DM, Javaherian A, Skibinski G, Lipnick S, et al. In Silico Labeling: Predicting Fluorescent Labels in Unlabeled Images. Cell. 2018;173(3):792–803.e19. doi: 10.1016/j.cell.2018.03.040 29656897 PMC6309178

[36] Cross-Zamirski JO, Mouchet E, Williams G, Schönlieb CB, Turkki R, Wang Y. Label-free prediction of cell painting from brightfield images. Scientific Reports. 2022;12(1):10001. doi: 10.1038/s41598-022-12914-x 35705591 PMC9200748

[37] Wieslander H, Gupta A, Bergman E, Hallström E, Harrison PJ. Learning to see colours: Biologically relevant virtual staining for adipocyte cell images. PLOS ONE. 2021;16(10):e0258546. doi: 10.1371/journal.pone.0258546 34653209 PMC8519425

