# Supplementary material for: Evaluating the utility of brightfield image data for mechanism of action prediction
Source: PLoS Comput Biol. 2023 Jul 25;19(7):e1011323. doi: 10.1371/journal.pcbi.1011323 (PMC10403126; doi:10.1371/journal.pcbi.1011323)
Supplement: S1 Text — Includes information on the cell culture, compounds, cell painting, image acquisition, CellProfiler feature extraction and the grit scores. (PDF) [file pcbi.1011323.s001.pdf]

Evaluating the utility of brightfield image data for mechanism of action prediction

S1 Text: Data acquisition

Cell culture: The human osteosarcoma cell line U2OS (ATCC; HTB-96) was cultured in Dulbecco's Minimum Essential Media (Gibco cat. no. 31885023) supplemented with 10% (v/v) fetal bovine serum (Gibco cat. no. 10500064), and 100 U/ml penicillin, and 100 μg/ml streptomycin (Gibco cat. no. 15140122). Cells were kept in a 37 °C humidified incubator with 5% CO2 atmosphere. We confirmed that the U2OS cell line was free from mycoplasma using the luminescence-based MycoAlert kit (Lonza cat. no. LT07-218).

Compounds: Compound handling was performed by the SciLifeLab Compound Center (CBCS, Solna, Stockholm). In brief, chemicals were solubilized in DMSO at a concentration of 10μM, then 40nl of each compound was dispersed using the Echo liquid handler into Falcon optilux microplates (Falcon, cat. no. BD353962) and stored at -20 °C prior to experimentation. Compounds were distributed over the plates with three technical replicates and two biological replicates. To reduce bias by positional effects in the microwell plates, the conditions were distributed over the plates using PLAID (Plate Layouts using Artificial Intelligence Design, [1]).

Cell Painting: The Cell Painting protocol [2] was followed with a few adjustments. A Biotek MultiFlo FX was used for dispensing cells and solutions and a Biotek 405 LS microplate washer was used for washing steps. A robotic arm (UR3) moves plates between the incubator, plate hotel, and washer and dispenser, using tailored software for scheduling (more info: https://github.com/pharmbio/aros). In short, 40 μl of cells were dispensed on top of DMSO solubilized compounds at a density of 1100 cells/well. The plates were incubated for 48 hr at 37 °C at 5% CO2 atmosphere. Then, assay plates were washed with 80 μl 1x PBS (Thermo Fisher, cat.no 11510546), followed by addition of 30 μl MitoTracker (Invitrogen; M22426) in prewarmed Live Cell Imaging solution (900nM). After 20 minutes of incubation, the cells were washed with 80 μl 1x PBS and fixed in 80 μl of 4% PFA (Histolab; 02176) for 20 min. The plates were washed three times, followed by permeabilization with 80 μl of 0.1% Triton X-100 for 20 min at room temperature and washed three times with PBS. Then, 20 μl staining mixture was added to each well reaching a final well-concentration of 10 μg/ml Hoechst, 15 μg/ml Wheat germ agglutinin, 5 μl/ml Phalloidin, 5 μM SYTO 14 and 40 μg/ml Concanavalin A, and was incubated for 20 min. The targets for each of the stains were: DNA (Hoechst); mitochondria (MitoTracker); Golgi apparatus and plasma membrane (Wheat Germ Agglutinin); F-actin (Phalloidin); nucleoli and cytoplasmic RNA (SYTO 14); and the endoplasmic reticulum (Concanavalin A/Alexa Fluor 488). Stains were removed and plates were washed three times with 1X PBS prior to imaging.

Image acquisition: Microplates were imaged using a widefield high-throughput ImageXpress Micro XLS (Molecular Devices) microscope with a 20X objective with laser-based autofocus. Fluorescent images were captured using five fluorescent channels. Excitation spectra were set to 377/50 nm (Hoechst), 628/40 nm (Mitotracker), 562/40 nm (Phalloidin and Wheat germ agglutinin), 531/40 nm (SYTO 14) and 482/35 nm (Concanavalin A). Emission filters were set to detect signals between 447/60 nm (Hoechst), 692/40 nm (MitoTracker), 624/40 nm (Wheat Germ Agglutinin and Phalloidin), 593/40 nm (SYTO 14) and 536/35 (Concanavalin A). For each well, a total

of nine fields of view were captured using a single z-plane targeting the cell compartment of interest. Brightfield images were captured under transmitted light, for five fields of views and 6 focal planes, 2μm apart from each other. The Hoechst staining was used for autofocus. Images were saved as 16-bit grayscale TIFF files without binning (2160x2160 pixels).

CellProfiler feature extraction: Morphological features were extracted with the open-source image analysis software CellProfiler version 4.0.6, CellPose generalist algorithm was used for cellular segmentation [3, 4]. Mean profiles were computed for all features on an image level. Varying and outlier features (SD < 0.001 and SD > 10000), as well as features with missing values were removed. For visualization of the affected features in radar plots, the absolute mean of the z-score normalized features was computed, grouped by Cell Profiler module, i.e. Intensity (I), Correlation (C), Granularity (G), Location (L) and RadialDistribution (RD); as well as by stains, i.e. Nucleus (Hoechst), ER (Concanavalin A), Nucleoli and cytoplasmic RNA (SYTO14), Golgi apparatus and F-actin cytoskeleton (WGA and Phalloidin) and Mitochondria (Mitotracker). Area-shape related features were grouped by cell compartment, i.e. Cell (C), Cytoplasm (Cy) and Nucleus (N). Neighboring related features were grouped by Cell (C) and Nucleus (N) and Neighbour features were grouped per cell compartment, i.e. Correlation features among the different stains were represented by their corresponding color code.

Grit scores: For assessing the reproducibility of a compound treatment and its perturbation strength (morphological difference) relative to a DMSO control, one can compute a grit score (`https://github.com/cytomining/cytominer-eval`, [5]). Based on CP features (extracted for the nuclei, cytoplasm and the entire cells in the FL images) we computed the grit scores for all the imaging sites used. A grit score of three for an imaging site means that on average the site is three standard deviations more similar to replicate sites for the same compound than it is to DMSO controls. The grit scores for some compounds (4 out of 231 compounds) were not calculated due to the images failing quality control, such as no cells present and out-of-focus images, and hence were not included in the grit-based analysis.

References

1. Francisco Rodríguez MA, Carreras Puigvert J, Spjuth O. Designing microplate layouts using artificial intelligence. Artificial Intelligence in the Life Sciences. 2023; p. 100073. doi:10.1016/j.ailsci.2023.100073.

2. Bray MA, Singh S, Han H, Davis CT, Borgeson B, Hartland C, et al. Cell Painting, a high-content image-based assay for morphological profiling using multiplexed fluorescent dyes. Nature protocols. 2016;11(9):1757–1774.

3. Stirling DR, Swain-Bowden MJ, Lucas AM, Carpenter AE, Cimini BA, Goodman A. CellProfiler 4: improvements in speed, utility and usability. BMC bioinformatics. 2021;22(1):1–11.

4. Stringer C, Wang T, Michaelos M, Pachitariu M. Cellpose: a generalist algorithm for cellular segmentation. Nature methods. 2021;18(1):100–106.

5. Trapotsi MA, Mouchet E, Williams G, Monteverde T, Juhani K, Turkki R, et al. Cell Morphological Profiling Enables High-Throughput Screening for PROteolysis TArgeting Chimera (PROTAC) Phenotypic Signature. ACS Chemical Biology. 2022;17(7):1733–1744. doi:10.1021/acschembio.2c00076.
