# Supplementary material for: Evaluating the utility of brightfield image data for mechanism of action prediction
Source: PLoS Comput Biol. 2023 Jul 25;19(7):e1011323. doi: 10.1371/journal.pcbi.1011323 (PMC10403126; doi:10.1371/journal.pcbi.1011323)
Supplement: S2 Text — Additional information on the model training strategy (optimizers, learning rate and augmentations applied). (PDF) [file pcbi.1011323.s002.pdf]

Evaluating the utility of brightfield image data for mechanism of action prediction

S2 Text: Model training

During training, the models were optimized with stochastic gradient descent (SGD), with a momentum of 0.9 and weight decay of 1e-4. The models were trained for 750 epochs with a batch size of 64. A linear warmup cosine annealing learning rate scheduler was applied, whereby the learning rate was increased linearly from 0.005 to 0.05 for the first 10 epochs and then reduced with cosine annealing to 5e-5 at the end. Class weights were used with the cross entropy loss function to account for imbalance in the number of compounds for each MoA. Random cropping (1024x1024 pixels) followed by resizing (to 512x512 pixels), grid shuffle, flipping, and 90-degree rotation augmentations were also used. The final model saved for inference was selected based on the accuracy for the validation data. All the experiments were run with a fixed random seed and all the hyperparameters were kept the same for both the BF and FL models.
