# Supplementary figures and images for: Evaluating the utility of brightfield image data for mechanism of action prediction
Source: PLoS Comput Biol. 2023 Jul 25;19(7):e1011323. doi: 10.1371/journal.pcbi.1011323 (PMC10403126; doi:10.1371/journal.pcbi.1011323)

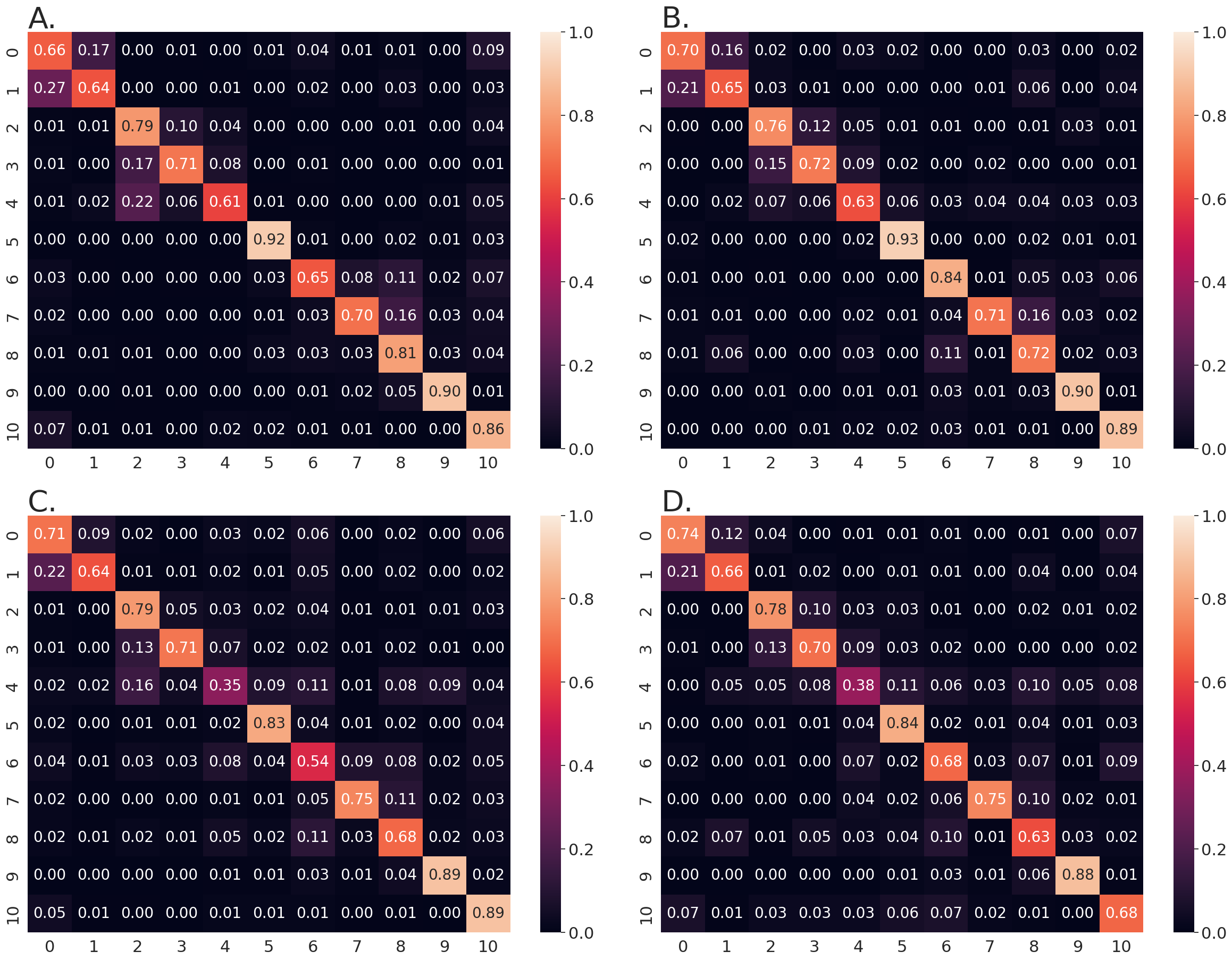

Supplement: S1 Fig — Normalized confusion matrices, across all five test sets, when DMSO plate-level normalization was applied to the data (A. BF models; B. FL models) and when the data was normalized at the imaging site level (C. BF models; D. FL models). Labels are 0:ATPase-i, 1:AuroraK-i, 2:HDAC-i, 3:HSP-i, 4:JAK-i, 5:PARP-i, 6:Prot.Synth.-i, 7:Ret.Rec.Ag, 8:Topo.-i, 9:Tub.Pol.-i, 10:DMSO. (PNG) [file pcbi.1011323.s003.png]

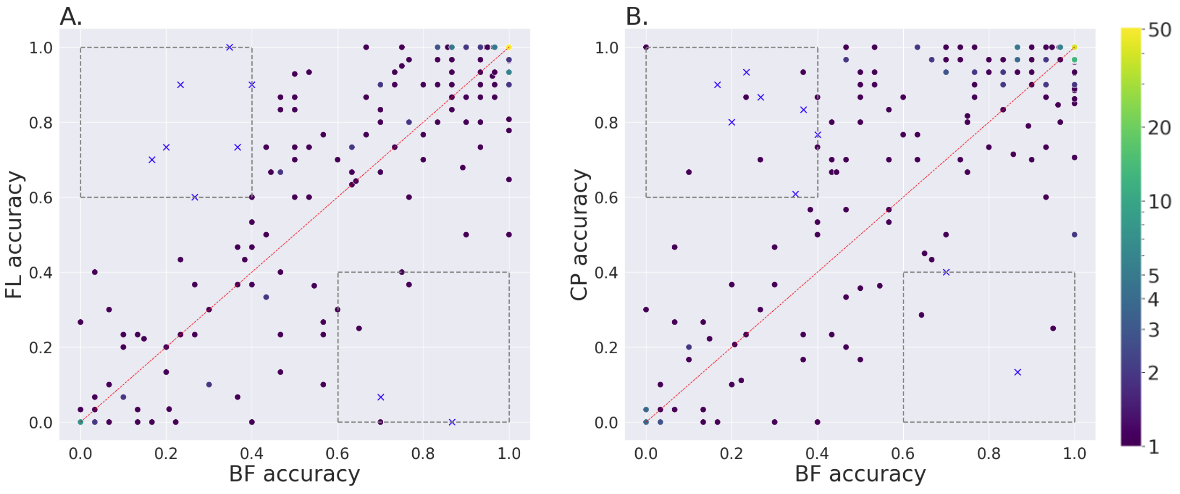

Supplement: S2 Fig — Comparison of the accuracy at the compound level, across all five test sets, for the BF models with respect to FL models, and CP feature-based models. The input data was normalized separately for each imaging site for BF and FL and was normalized based on the DMSO on each plate for CP. Each dark dot represents a compound. Brighter dots represent multiple compounds with the same accuracy score. A. BF against FL; B. BF against CP. In the boxes at the bottom right and top left, thresholded at accuracy values of 0.6 and 0.4, the compounds shown with blue crosses were consistently better for BF than both FL and CP or consistently worse, respectively. (PNG) [file pcbi.1011323.s004.png]

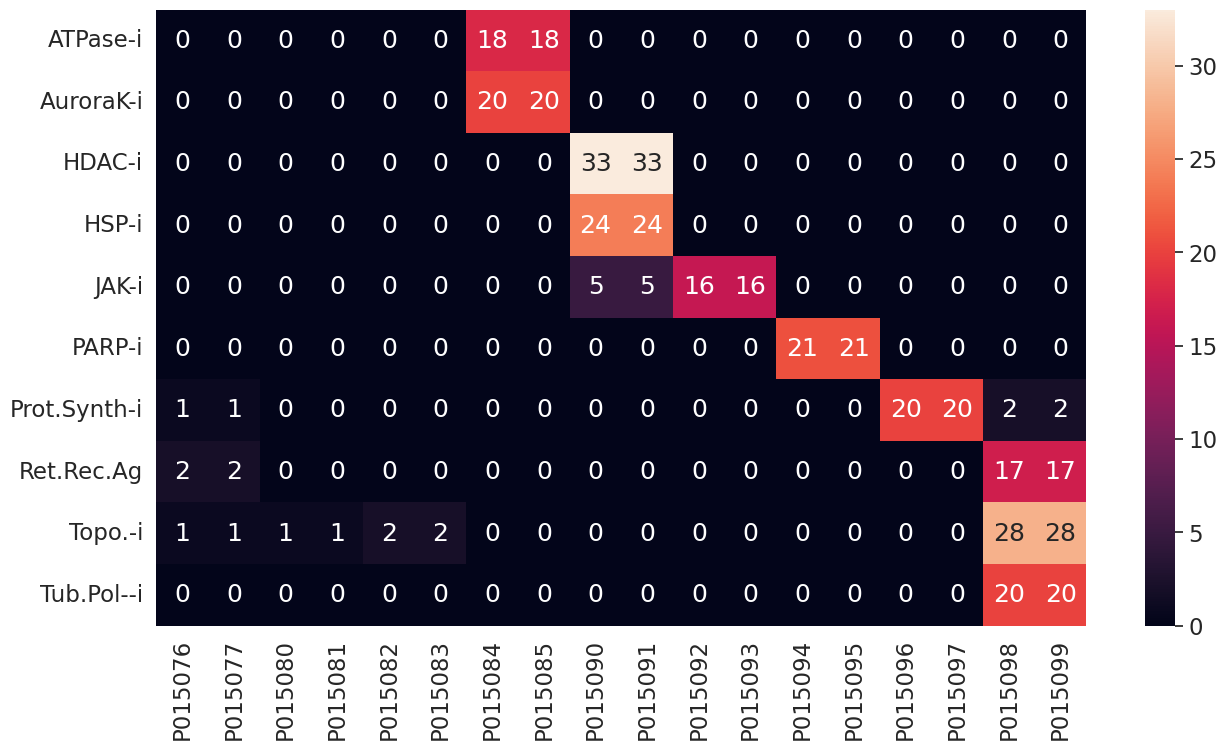

Supplement: S3 Fig — The number of compounds per MoA across each of the imaging plates used in our study. Note that there are pairs of plates, biological replicates, with the same compound treatments, but the treatments were located in different wells within each of the replicate plates. (PNG) [file pcbi.1011323.s005.png]

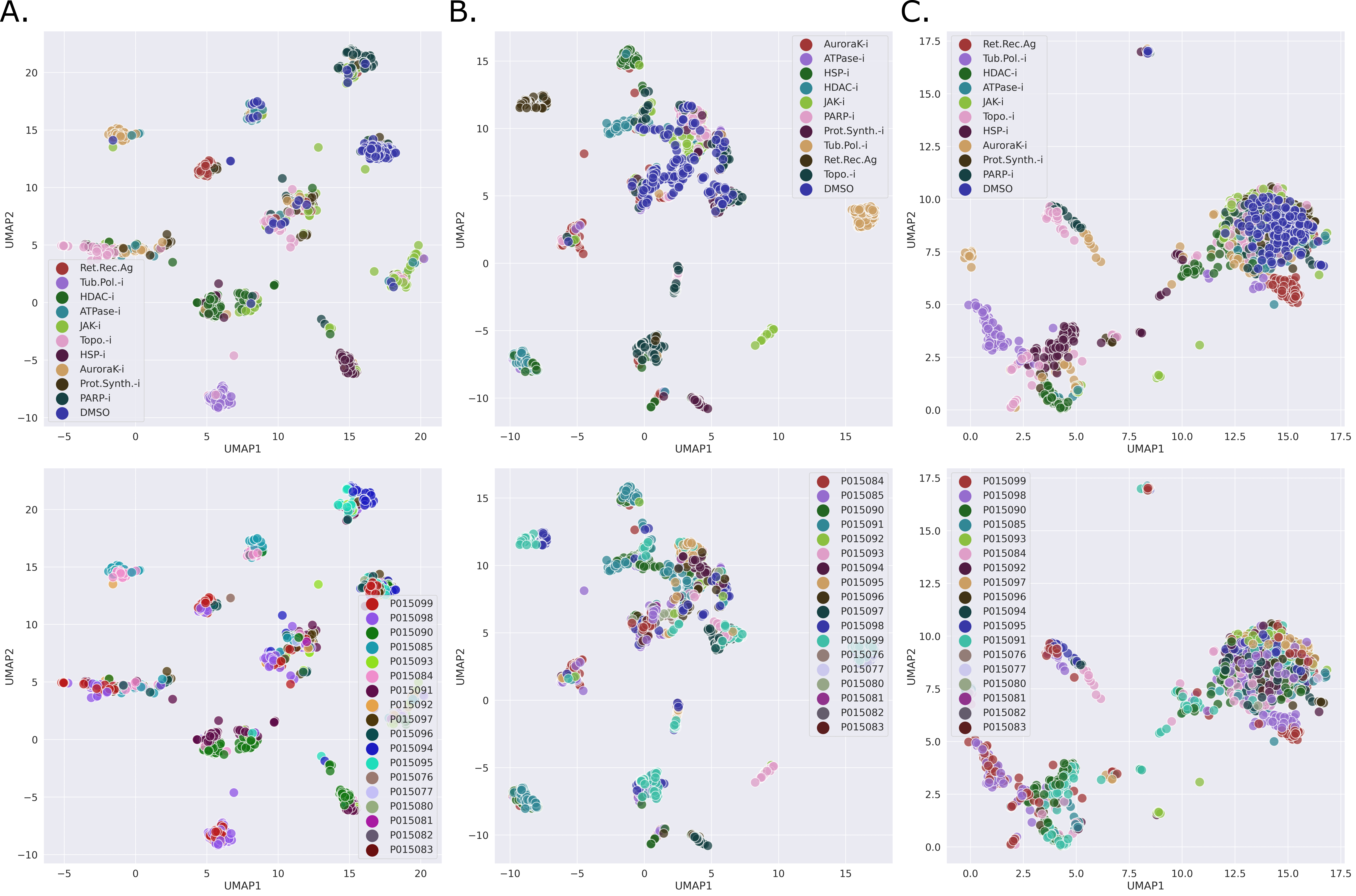

Supplement: S4 Fig — UMAP plots of features learned by the BF and FL models (based on site-level normalization of the input images), and the raw CP features of the test set for the best-performing split (split 2), colour-coded by MoA (top-row) and by plate (bottom-row). The UMAP was fitted on the training data of the split and the test set features were transformed. A. BF features; B. FL features; C. CP cell-based features. (PNG) [file pcbi.1011323.s006.png]

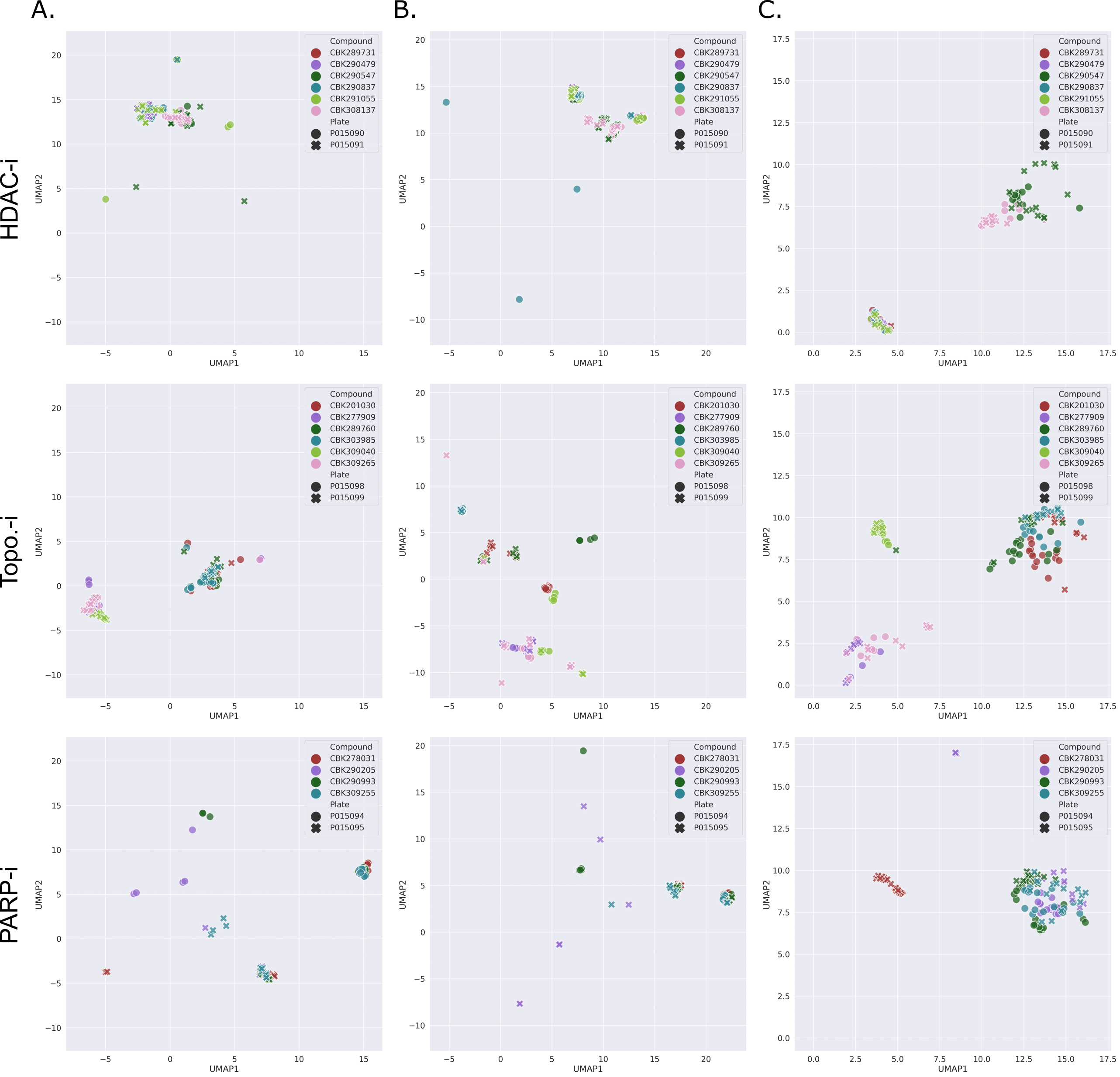

Supplement: S5 Fig — UMAP plots of features learned by the BF and FL models (based on DMSO normalization of the input images), and the raw CP features of the test set for the best-performing split (split 2) for three of the MoAs, color coded by compound and shape coded by plate. A. BF features; B. FL features; C. CP cell-based features. (PNG) [file pcbi.1011323.s007.png]

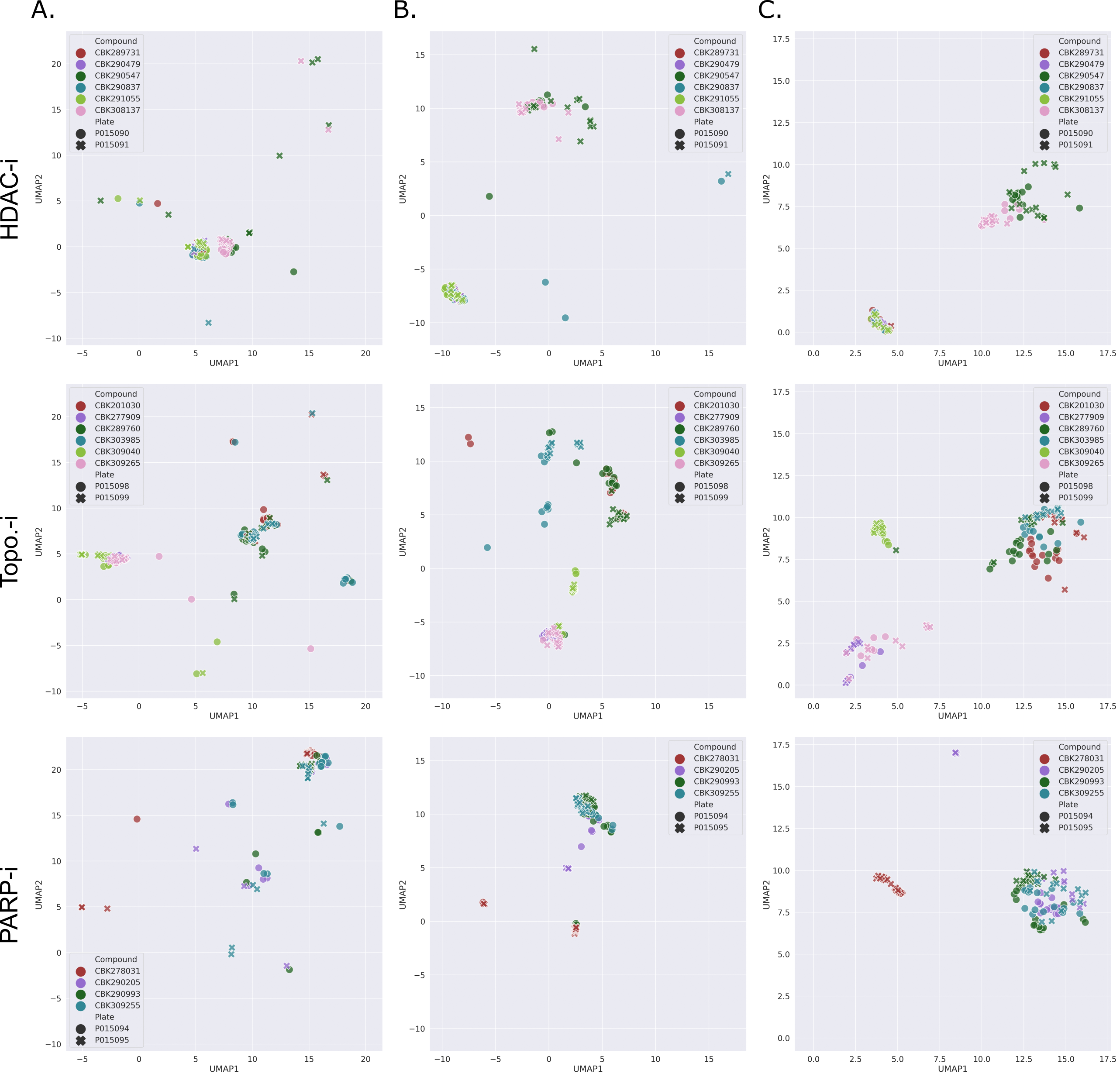

Supplement: S6 Fig — UMAP plots of features learned by the BF and FL models (based on site-level normalization of the input images), and the raw CP features of the test set for the best-performing split (split 2) for three of the MoAs, color coded by compound and shape coded by plate. A. BF features; B. FL features; C. CP cell-based features. (PNG) [file pcbi.1011323.s008.png]

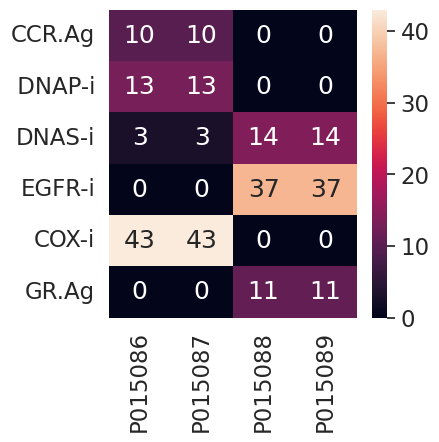

Supplement: S7 Fig — The number of compounds per MoA across each of the imaging plates used in our downstream analysis. Note that there are pairs of plates, biological replicates, with the same compound treatments, but the treatments were located in different wells within each of the replicate plates. (PNG) [file pcbi.1011323.s009.png]
