# Supplementary material for: Evaluating the utility of brightfield image data for mechanism of action prediction
Source: PLoS Comput Biol. 2023 Jul 25;19(7):e1011323. doi: 10.1371/journal.pcbi.1011323 (PMC10403126; doi:10.1371/journal.pcbi.1011323)
Supplement: S1 Table — Comparison of the results for three model runs with different seeds when DMSO plate-level normalization was applied to the data. (PDF) [file pcbi.1011323.s010.pdf]

Evaluating the utility of brightfield image data for mechanism of action prediction

Table S1. Comparison of the results for three model runs with different seeds when DMSO plate-level normalization was applied to the data.

(a) Mean and standard deviation of the Macro-F1 scores on the test sets for the five data splits.

	BF_{dmso}	FL_{dmso}
Split 1	0.734±0.005	**0.765±0.009**
Split 2	**0.819±0.004**	0.807±0.006
Split 3	0.723±0.001	**0.782±0.008**
Split 4	0.689±0.015	**0.744±0.008**
Split 5	0.720±0.009	**0.743±0.020**

(b) Mean and standard deviation of the F1 scores per MoA on the test sets.

	BF_{dmso}	FL_{dmso}
ATPase-i	0.622±0.012	**0.728±0.020**
AuroraK-i	0.681±0.002	**0.711±0.028**
HDAC-i	0.753±0.003	**0.786±0.011**
HSP-i	**0.741±0.009**	0.720±0.011
JAK-i	0.662±0.010	**0.668±0.010**
PARP-i	0.889±0.005	**0.905±0.014**
Prot.Synth.-i	0.682±0.021	**0.772±0.015**
Ret.Rec.Ag	0.743±0.005	**0.764±0.004**
Topo.-i	**0.755±0.020**	0.719±0.007
Tub.Pol.-i	**0.882±0.005**	0.857±0.003
DMSO	0.773±0.013	**0.867±0.006**
Macro average	0.744±0.005	**0.772±0.005**
