# Supplementary material for: Evaluating the utility of brightfield image data for mechanism of action prediction
Source: PLoS Comput Biol. 2023 Jul 25;19(7):e1011323. doi: 10.1371/journal.pcbi.1011323 (PMC10403126; doi:10.1371/journal.pcbi.1011323)
Supplement: S2 Table — Comparison of the results for three model runs with different seeds when site-level normalization was applied to the data. (PDF) [file pcbi.1011323.s011.pdf]

Evaluating the utility of brightfield image data for mechanism of action prediction

Table S2. Comparison of the results for three model runs with different seeds when site-level normalization was applied to the data.

(a) Mean and standard deviation of the Macro-F1 scores on the test sets for the five data splits.

	BF_{site}	FL_{site}
Split 1	0.661±0.004	**0.644±0.022**
Split 2	**0.772±0.003**	0.750±0.010
Split 3	0.657±0.002	**0.668±0.007**
Split 4	**0.673±0.006**	0.650±0.008
Split 5	**0.706±0.004**	0.691±0.002

(b) Mean and standard deviation of the F1 scores per MoA on the test sets.

	BF_{site}	FL_{site}
ATPase-i	0.657±0.007	**0.677±0.004**
AuroraK-i	**0.714±0.001**	0.667±0.003
HDAC-i	0.766±0.002	**0.786±0.003**
HSP-i	**0.751±0.004**	0.692±0.008
JAK-i	0.378±0.025	**0.407±0.022**
PARP-i	**0.781±0.007**	0.735±0.012
Prot.Synth.-i	0.517±0.008	**0.634±0.010**
Ret.Rec.Ag	0.785±0.013	**0.791±0.016**
Topo.-i	**0.687±0.011**	0.643±0.007
Tub.Pol.-i	0.858±0.006	**0.874±0.006**
DMSO	**0.846±0.007**	0.684±0.007
Macro average	**0.704±0.001**	0.690±0.005
