# Supplementary material for: Evaluating the utility of brightfield image data for mechanism of action prediction
Source: PLoS Comput Biol. 2023 Jul 25;19(7):e1011323. doi: 10.1371/journal.pcbi.1011323 (PMC10403126; doi:10.1371/journal.pcbi.1011323)
Supplement: S3 Table — Comparison of the test set F1 scores for three different architectures trained on BF and FL when DMSO plate-level normalization was applied to the data. (PDF) [file pcbi.1011323.s012.pdf]

Evaluating the utility of brightfield image data for mechanism of action prediction

Table S3. Comparison of the test set F1 scores for three different architectures trained on BF and FL when DMSO plate-level normalization was applied to the data.

(a) Macro-F1 scores on the test sets for the five data splits.

	ResNet-50		DenseNet-169		EfficientNet-B3	
	BF_{dmso}	FL_{dmso}	BF_{dmso}	FL_{dmso}	BF_{dmso}	FL_{dmso}
Split 1	0.738	**0.777**	0.726	0.768	0.749	0.758
Split 2	0.821	0.799	0.806	0.820	0.661	**0.836**
Split 3	0.724	0.793	0.710	0.760	0.301	**0.800**
Split 4	0.710	0.738	0.697	0.744	0.296	**0.785**
Split 5	0.728	0.716	0.711	0.755	0.436	**0.773**

(b) F1 scores per MoA on the test sets.

	ResNet-50		DenseNet-169		EfficientNet-B3	
	BF_{dmso}	FL_{dmso}	BF_{dmso}	FL_{dmso}	BF_{dmso}	FL_{dmso}
ATPase-i	0.605	0.701	0.591	0.719	0.398	**0.746**
AuroraK-i	0.683	0.675	0.663	0.708	0.538	**0.731**
HDAC-i	0.756	0.773	0.769	0.789	0.675	**0.797**
HSP-i	0.738	0.730	**0.743**	0.708	0.619	0.726
JAK-i	0.675	0.653	0.655	0.666	0.436	**0.698**
PARP-i	0.895	0.886	0.884	0.902	0.551	**0.935**
Prot.Synth.-i	0.711	0.793	0.701	0.778	0.296	**0.800**
Ret.Rec.Ag	0.740	0.769	0.744	0.776	0.510	**0.800**
Topo.-i	**0.780**	0.728	0.747	0.722	0.450	0.739
Tub.Pol.-i	**0.887**	0.854	0.847	0.868	0.735	0.843
DMSO	0.790	0.866	0.750	0.883	0.482	**0.897**
Macro average	0.751	0.766	0.736	0.774	0.517	**0.792**
